# Hydrogen–independent CO_2_ reduction dominates methanogenesis in five temperate lakes that differ in trophic states

**DOI:** 10.1093/ismeco/ycae089

**Published:** 2024-06-21

**Authors:** Dimitri Meier, Sigrid van Grinsven, Anja Michel, Philip Eickenbusch, Clemens Glombitza, Xingguo Han, Annika Fiskal, Stefano Bernasconi, Carsten J Schubert, Mark A Lever

**Affiliations:** Department of Environmental Systems Science, Institute of Biogeochemistry and Pollutant Dynamics, Swiss Federal Institute of Technology, Zurich (ETH Zurich), Universitätstrasse 16, 8092 Zurich, Switzerland; Ecological Microbiology, Bayreuth Center of Ecology and Environmental Research, University of Bayreuth, Dr. Hans-Frisch-Straße 1-3, 95448 Bayreuth, Germany; Department of Surface Waters—Research and Management, Swiss Federal Institute of Aquatic Science and Technology (EAWAG), Seestrasse 79, 6047 Kastanienbaum, Switzerland; Geomicrobiology, Department of Geosciences, Eberhard Karls Universität Tübingen (Tübingen University), Schnarrenbergstraße 94-96, 72076 Tübingen, Germany; Department of Environmental Systems Science, Institute of Biogeochemistry and Pollutant Dynamics, Swiss Federal Institute of Technology, Zurich (ETH Zurich), Universitätstrasse 16, 8092 Zurich, Switzerland; Department of Environmental Systems Science, Institute of Biogeochemistry and Pollutant Dynamics, Swiss Federal Institute of Technology, Zurich (ETH Zurich), Universitätstrasse 16, 8092 Zurich, Switzerland; Department of Environmental Systems Science, Institute of Biogeochemistry and Pollutant Dynamics, Swiss Federal Institute of Technology, Zurich (ETH Zurich), Universitätstrasse 16, 8092 Zurich, Switzerland; Department of Environmental Systems Science, Institute of Biogeochemistry and Pollutant Dynamics, Swiss Federal Institute of Technology, Zurich (ETH Zurich), Universitätstrasse 16, 8092 Zurich, Switzerland; Department of Environmental Systems Science, Institute of Biogeochemistry and Pollutant Dynamics, Swiss Federal Institute of Technology, Zurich (ETH Zurich), Universitätstrasse 16, 8092 Zurich, Switzerland; Department of Earth Sciences, Swiss Federal Institute of Technology, Zurich (ETH Zurich), Geological Institute, Sonneggstrasse 5, 8092 Zurich, Switzerland; Department of Environmental Systems Science, Institute of Biogeochemistry and Pollutant Dynamics, Swiss Federal Institute of Technology, Zurich (ETH Zurich), Universitätstrasse 16, 8092 Zurich, Switzerland; Department of Surface Waters—Research and Management, Swiss Federal Institute of Aquatic Science and Technology (EAWAG), Seestrasse 79, 6047 Kastanienbaum, Switzerland; Department of Environmental Systems Science, Institute of Biogeochemistry and Pollutant Dynamics, Swiss Federal Institute of Technology, Zurich (ETH Zurich), Universitätstrasse 16, 8092 Zurich, Switzerland; Marine Science Institute, Department of Marine Sciences, University of Texas at Austin, 750 Channel View Drive, Port Aransas, TX 78373, United States

**Keywords:** archaeal methanogenesis, methane oxidation, hydrogenotrophic, aceticlastic, methylotrophic, direct interspecies electron transfer (DIET), eutrophication, sediment

## Abstract

Emissions of microbially produced methane (CH_4_) from lake sediments are a major source of this potent greenhouse gas to the atmosphere. The rates of CH_4_ production and emission are believed to be influenced by electron acceptor distributions and organic carbon contents, which in turn are affected by anthropogenic inputs of nutrients leading to eutrophication. Here, we investigate how eutrophication influences the abundance and community structure of CH_4_ producing *Archaea* and methanogenesis pathways across time–resolved sedimentary records of five Swiss lakes with well–characterized trophic histories. Despite higher CH_4_ concentrations which suggest higher methanogenic activity in sediments of eutrophic lakes, abundances of methanogens were highest in oligotrophic lake sediments. Moreover, while the methanogenic community composition differed significantly at the lowest taxonomic levels (OTU), depending on whether sediment layers had been deposited under oligotrophic or eutrophic conditions, it showed no clear trend in relation to *in situ* distributions of electron acceptors. Remarkably, even though methanogenesis from CO_2_-reduction was the dominant pathway in all sediments based on carbon isotope fractionation values, taxonomic identities, and genomes of resident methanogens, CO_2_-reduction with hydrogen (H_2_) was thermodynamically unfavorable based on measured reactant and product concentrations. Instead, strong correlations between genomic abundances of CO_2_-reducing methanogens and anaerobic bacteria with potential for extracellular electron transfer suggest that methanogenic CO_2_-reduction in lake sediments is largely powered by direct electron transfer from syntrophic bacteria without involvement of H_2_ as an electron shuttle.

## Introduction

Lake sediments are globally important organic carbon (OC) sinks that, despite the much smaller surface area of lakes, have annual OC burial rates in the range of marine sediments [1, 2]. In addition, lake sediments are important sources of greenhouse gasses, such as carbon dioxide (CO_2_) and methane (CH_4_) [3, 4]. CH_4_ release from lakes is on the rise due to human activities that increase OC loads (eutrophication), raise water temperatures, and promote bottom-water oxygen (O_2_) depletion [5–7]. It has been estimated that freshwater sediments are the biggest natural source of CH_4_ to the atmosphere (~122 Tg CH_4_ yr^−1^) after freshwater wetlands (~185 Tg CH_4_ yr^−1^) and exceed total emissions from oceanic sources (~14 Tg CH_4_ yr^−1^) [8].

Most of the CH_4_ in lake sediments is generated by anaerobic *Archaea*, also known as “methanogens”. Methanogenic *Archaea* gain energy by converting end products of the fermentative degradation of organic matter, e.g. H_2_ + CO_2_, acetate, and methanol, to CH_4_ [9]. The distribution of methanogenic activity is in part controlled by competition with other anaerobic microorganisms that respire nitrate, metal oxides (Mn(IV), Fe(III)), or sulfate. Because these electron acceptors typically provide higher energy gains from the same substrates [10], methanogens often only dominate respiration in deeper layers (“methanogenesis zones”), where competing electron acceptors have been depleted.

Multiple archaeal taxa have been linked to CH_4_ production. Among these, the euryarchaeotal orders *Methanomicrobiales*, *Methanosarcinales*, *Methanocellales*, and *Methanobacteriales* are often considered to be the main CH_4_ producers in lake sediments [11]. Yet, other, more recently discovered groups such as the euryarchaeotal order *Methanomassiliicoccales* [12, 13] and class *Methanonatronarchaeia* [14], as well as putative methanogens identified by metagenomic analyses [15–17] could also be important. In addition, several environmental clusters of *mcr*A, a well-studied marker gene of CH_4_-cycling *Archaea* that encodes the alpha subunit of methyl-coenzyme M reductase, remain phylogenetically unassigned [18].

Archaeal methanogenesis is known to proceed via four pathways: CO_2_ reduction with H_2_ or formate as electron donors (“hydrogenotrophic”), acetate disproportionation (“aceticlastic”), dismutation of methylated compounds, e.g. methanol, methyl amines, or methyl sulfides, with or without H_2_ (“methylotrophic”; for review, see [9]), and O-demethylation of methoxylated compounds (methoxydotrophic; [19]). Among these, acetate disproportionation and CO_2_ reduction are considered the dominant pathways on Earth with theoretical calculations predicting a 2:1 ratio of the former to the latter [11, 20]. Yet, carbon stable isotopic data paint a more complex picture, e.g. dominance of CO_2_ reduction in marine sediments versus dominance of aceticlastic methanogenesis in freshwater sediments [21]. However, even in freshwater lakes, isotopic data and methanogenic community composition indicate CO_2_ reduction to dominate in some cases [20, 22, 23].

One potential driver of methanogenic pathways in nature is the chemical composition of organic matter from which methanogenic substrates are produced. Fermentation reactions, which are the main sources of methanogenic substrates, vary in the compositions of their end products based on the chemical structure of the compounds that are being fermented. For instance, methanol is known to be released during the degradation of aromatic acids and pectin from vascular plants [24, 25], whereas acetate may mainly form from fresh or labile algal organic matter [26]. Based on these past studies, one might speculate that the process of eutrophication, which promotes phytoplankton growth and sedimentation of labile algal organic matter, should favor aceticlastic methanogenesis, while high inputs of terrestrial OC should lead to elevated contributions of methylotrophic methanogenesis. Yet, the existing data do not necessarily support this view and suggest additional important roles of temperature and interactions with syntrophic bacteria [11]. Adding to the complexity, laboratory studies show that certain aceticlastic *Methanosarcinales* (*Methanosarcina* and *Methanothrix*) can reduce CO_2_ to CH_4_ using electrons from electrogenic partner organisms [27]. The potential for direct electron transfer between syntrophic bacteria and methanogens in lake sediments was subsequently shown by adding conductive particles to anoxic sediment slurries [28, 29], but the environmental significance of this process remains unknown.

Understanding the drivers behind methanogenic pathways is key to understanding the lacustrine carbon cycle and how it will respond to future anthropogenic perturbations. Here, we investigate the long-term impact of eutrophication on dominant methanogenic pathways and on the abundance and community structure of methanogenic *Archaea* across time–resolved sedimentary records of five lakes in central Switzerland. Carbon isotopic values of CH_4_ and dissolved inorganic carbon (DIC) and taxonomic compositions of methanogens indicate CO_2_ reduction as the main methanogenic pathway. Yet, Gibbs energies of methanogenic CO_2_ reduction with H_2_, based on measured substrate and product concentrations in natural sediments, suggest this pathway to be largely endergonic and unlikely to be important. Complementary metagenomic analyses, which indicate significant correlations between abundances of CO_2_-reducing methanogens and syntrophic bacteria with potential for extracellular electron transfer (EET), lead us to propose that electrotrophic CO_2_ reduction is the dominant methanogenic pathway in the lakes studied.

## Materials and methods

### Lake trophic histories

All five lakes are well-characterized with respect to their trophic histories. (Table 1; for details see [6]). After the era of peak eutrophication (~1950 to 1970), P bans on detergents and improved wastewater management lowered P inputs significantly [6]. As a result, Lake Zurich returned to a mesotrophic state in ~1980, though the deep basin of this lake remains hypoxic today. By contrast, Lake Greifen, Lake Baldegg, and Lake Zug remain eutrophic due to retention of P that was introduced in the 20th century. Despite artificial water column mixing and aeration (Lake Baldegg: since 1982/83; Lake Greifen; since 2009), Lake Baldegg experiences strong water column oxygen decreases and Lake Greifen becomes hypoxic or anoxic below 10 m water depth every summer. By contrast, Lake Lucerne, despite slight increases in P concentrations in the 1960s and 1970s, always remained oligotrophic.

**Table 1:** Water depths of sites and trophic histories of the five investigated lakes.

Lake	Water depth (m)			Onset of eutrophication (year)	Present-day trophic state
	**Shallow station**	**Medium station**	**Deep station**		
Greifen	15	24	32	~1920	Eutrophic
Baldegg	21	45	66	~1890	Eutrophic
Zug	25	35	50	~1930	Eutrophic
Zurich	25	45	137	~1890	Mesotrophic (since 1980)
Lucerne	24	45	93	–	Oligotrophic

### Sampling and *in situ* biogeochemical zonation

The long-term influence of eutrophication on the sedimentary carbon cycle in the five lakes has been studied as part of the research effort “Lake Eutrophication Impacts on Carbon Accumulations in Sediments”. During a field campaign in June/July 2016, three stations in each lake that differed in water depths (“shallow”, “medium”, “deep”) were sampled using UWITEC gravity corers with 15-cm liner diameters (Table 1; also see [6]). Detailed analyses of biogeochemical gradients, macrofaunal communities, and microbial community structure have been performed and complemented by high-resolution sediment age models, with which the trophic state at the time of deposition could be determined for sediment layers throughout these cores [6, 30, 31].

In all lakes, bottom water temperatures at the time of sampling ranged from 5 to 9°C and the pH of sediment pore water was neutral to slightly alkaline (7.0 to 8.4). O_2_ was depleted in the top 1 cm, with the shallowest penetrations (average ± standard deviation (SD)) in the hypoxic deep basin of Lake Zurich (no measurable O_2_), Lake Baldegg (0.08 ± 0.02 cm), and Lake Greifen (0.17 ± 0.03 cm), and the deepest penetration in Lake Lucerne (0.73 ± 0.25 cm). Notably, the dominant anaerobic respiration reactions overlapped in all lakes, with methanogenesis setting in at sediment depths where the reduction of nitrate, sulfate, iron(III) and manganese(IV) were still occurring [6, 32]. As a result, the methanogenesis zone started at the surface in Lakes Baldegg, Greifen and Zug, at 2.6 ± 2.4 cm in Lake Zurich, and 3.0 ± 1.7 cm in Lake Lucerne [6].

### Geochemical and isotopic analyses

CH_4_*and DIC.* CH_4_ and DIC concentration profiles and δ^13^C-CH_4_ were previously published [6, 30]. We here additionally measured δ^13^C-DIC compositions as described in [33]. Based on measured δ^13^C-CH_4_ and -DIC compositions, we calculate the fractionation factor α_C_ [34] using the equation:

α_C_ = (δ^13^C-DIC + 10^3^) / (δ^13^C-methane +10^3^).

Assuming minimal contributions of other methanogenic pathways, α_C_ > 1.065 indicates mainly CO_2_ reduction, while α_C_ < 1.055 indicates mainly aceticlastic methanogenesis.

*Dihydrogen (H_2_).* Concentrations of dissolved H_2_ were measured using the incubation method [35]. Sediment cores were extruded layer by layer (depth resolution: 0.5 cm from 0–2 cm sediment depth, 1 cm from 2–4 cm, 2 cm from 4–20 cm, 4 cm below 20 cm) and each depth layer was sampled immediately using sterile cut-off syringes. Two milliliters of sediment were quickly transferred to 20 mL headspace vials, where they were flushed for at least 1 minute to restore anoxic conditions, and then sealed with butyl rubber stoppers and crimped, prior to incubation at +4°C in the dark for 24 hrs (for further details, see Supplementary material). Two milliliters of headspace gas from each vial were then injected into a Peak Performer 1 (Peak Laboratories, USA) with a 1-mL sampling loop and a reducing compound photometer (RCP) equipped with a zeolite 81’ × 1/8” Molesieve 13X 60/80 column (Restek, USA) and a silica 1/8” × 16 ½” Unibeads 1S 60/80 (Grace, USA) column (RCP bed temperature: 265°C; column temperature: 105°C) using N_2_ as a carrier gas. Standard curves of H_2_ in He or N_2_ were used for one-point calibrations using a certified 4.7 ppm H_2_ in N_2_ gas standard (Linde, Germany). Headspace H_2_ concentrations were calculated based on the equation:

[H_2_]*_g_* = χH_2_ x P x R^−1^ x T^−1^.

where χH_2_ is the mol fraction of H_2_ (in parts per billion), P the headspace gas pressure (1 atm), R the universal gas constant (0.008314 kJ mol^−1^ K^−1^), and T the incubation temperature (in K). Dissolved H_2_ concentrations were calculated by multiplying [H_2_]*_g_* by the Bunsen coefficient, β, for freshwater at +4°C ([36]; β = 0.02081).

*Volatile fatty acids*: Pore water concentrations of volatile fatty acids (VFAs) were measured on a two-dimensional ion chromatography system as previously published [37] with minor modifications. The instrument used was a Dionex ICS6000+ system (Thermo Scientific). Separation was achieved with a 2-mm AS24 column for the first dimension and a 2-mm AS11HC column for the second dimension. The retention time window for collecting VFAs from the first chromatographic dimension was set to 3–6.8 min. Quantification was achieved by conductivity detection at detection limits of 0.1–0.4 μM (for details see [37]). Prior to analysis, 1 mL of sample pore water was filtered through Milli-Q washed IC grade syringe filters (Acrodisc, Supor® membrane, 13 mm diameter). The first 0.5 mL of the filtered pore water was discarded, while the second 0.5 mL was used for the analysis.

### Thermodynamic calculations

*In situ* Gibbs energy yields (*ΔG_r_’)* of methanogenesis reactions from H_2_ + CO_2_, acetate, methanol, and methanol+H_2_ were calculated based on the equation:

ΔG*_r_*’ = ΔG*_r_*^0^_(TP)_ + RT ln Q*_r_.*

where *ΔG_r_^0^_(TP)_* is the Gibbs energy (kJ mol^−1^ of reaction) at standard concentrations (1 M per each reactant and product, pH 7.0) at *in situ* temperature T (K) and pressure P (bar), R is the universal gas constant (0.008314 kJ mol^−1^ K^−1^), and Q*_r_* the quotient of (except where noted otherwise) measured product and reactant activities. The standard Gibbs energy of the reaction *ΔG_r_^0^* was corrected to *in situ* temperature and pressure as outlined in [38]. Standard Gibbs energies, enthalpies, and molal volumes of formation are shown in Table S1. Activities of aqueous species were calculated using the activity coefficients γ_HCO3-_ = 0.832 (calculated using PHREEQC v3) and γ_CH4_ = 1.24 [39]. The activity coefficient of H_2_ was assumed to equal that of CH_4_, whereas activities of acetate and methanol were assumed to equal 1.0. All concentrations were measured as described above except those of methanol. For methanol we conservatively assumed a concentration of 1 nM (typical methanol concentrations in sediments are higher (0.1 to 1 μM); [40]).

### Analysis of methanogenic community structure

*DNA extraction.* DNA was extracted following the modular extraction method of [41]. For the exact protocol used, see [31].

*Quantitative PCR.* The primer combination mlas F (5′- GGT GTM GGD TTC ACM CAR TA)—mcrA-rev (5′-CGT TCA TBG CGT AGT TVG GRT AGT) was used for *mcr*A quantification by quantitative polymerase chain reaction (qPCR). Each reaction was run in triplicate and consisted of a final volume of 10 μL (5 μL SYBR Green I Master Mix (Roche Molecular Systems), 0.5 μL of each 10 μM primer solution, 1 μL 10 mg mL^−1^ bovine serum albumin, 2 μL DNA extract, 1 μL molecular grade water). qPCR assays were performed on a LightCycler 480 II (Roche Molecular Systems) and consisted of (i) enzyme activation: 95°C, 5 min; (ii) 50 cycles of (a) denaturation, 95°C, 10 s, (b) annealing: 53°C, 20 s, (c) elongation: 72°C, 30 s, and (d) fluorescence measurement: 84°C, 5 s; and (iii) stepwise melting curve: 95°C to 53°C in 1 min intervals to check for primer specificity. qPCR standards consisted of a tenfold dilution series of complete *mcr*A genes of *Methanocorpusculum parvum* (~6.59 × 10^8^ copies μL). Contributions of CH_4_-cycling *Archaea* to total microbial communities were calculated based on ratios of *mcr*A copies to total 16S ribosomal ribonucleic acid (rRNA) gene copies from [31].

*Amplicon libraries.* Amplicon libraries were prepared as described in [31] and involved the same *mcr*A primer combination and amplication protocol as for qPCR. Sequencing was done on an Illumina MiSeq (Illumina Inc.). Raw sequence reads were quality-checked by *FastQC* (www.bioinformatics.babraham.ac.uk/projects/fastqc), read ends trimmed using *seqtk* (github.com/lh3/seqtk), paired end reads merged into amplicons by *FLASH* [42], primer sites trimmed by *usearch* [43], and quality filtering was done by *prinseq* [44]. Zero-radius Operational Taxonomic Units were generated using UNOISE3 [45] and clustered into OTUs using a 97% similarity threshold. These OTUs were taxonomically identified based on neighbor-joining phylogenetic trees in ARB using a publicly accessible *mcr*A database (*mcrA4All*; [46]). This database contained >2400 high-quality *mcrA* sequences from a wide range of published amplicon and whole-genome sequencing studies with manually optimized sequence alignments. Diversity calculations, sample ordination and statistical tests were performed in R using the vegan (v. 2.6-4) package [47]. Visualizations were done in R with the ggplot2 package [48] and esthetically edited in Inkscape vector graphics software.

### Analysis of metabolic potential of methanogens and partner organisms

#### Metagenome assembly and functional annotation

DNA extracted from five sediment layers of the deep station of each lake (25 in total) was shipped to the Joint Genome Institute (JGI), Walnut Creek, CA, USA, where it was sequenced and assembled according to the standard JGI pipeline (see Supplementary material for details). Criteria used to search for the reported genes are in SQL query log (File S1). Metagenomes from two additional sediment layers per lake were sequenced with the same technology at the Functional Genomics Center Zürich and used for read mapping to the annotated assemblies. For more detailed information, see the Supplementary material.

#### Metagenome-assembled genomes generation and classification

The 25 metagenome assemblies were subjected to differential-coverage and tetranucleotide–frequency-based binning and summarized into one non-redundant set of bins. Bins were analyzed for size, completeness, redundancy, and contig length, and manually refined to reduce “contamination”, i.e. duplications of single-copy genes (for details, see Supplementary material). After refinement, bins with a completeness of >50% and contamination <5% were considered for further analysis (434 in total). MAGs were taxonomically classified by GTDB-Tk [49]. For MAGs of *Methanomicrobia*, the classification was confirmed by calculating a *de novo* phylogenetic tree based on amino acid positions conserved in at least 25% of the concatenated sequence alignments of 53 marker proteins [50] made by GTDB-Tk [49]. The main metrics of the MAGs can be found in Table S2.

#### Identification of potential syntrophic partners of methanogens

Spearman rank correlations between coverage patterns of MAGs were calculated using cor.test function in R as implemented in [51]. Only MAGs with a coverage of >0.5x that were present in >20% of the samples were included. Among methanogens, only six MAGs (all *Methanoregulaceae*) fulfilled this criterion. MAGs showing a significant (*P* < .05; ρ > 0.75) to at least one methanogenic MAG were considered potential partner organisms (Table S3).

The true abundances of microbial taxa were estimated by multiplying relative abundances of the MAGs by qPCR-determined 16S gene copy numbers. Spearman rank correlations between estimated abundances of methanogens and other members of the microbial community were calculated as in [51]. MAGs showing a positive correlation coefficient of >0.75 to at least one methanogenic MAG were considered potential partner organisms.

#### Potential for extracellular electron transfer in MAGs of methanogens and syntrophic partners

Encoded proteins containing multiple heme-binding sites (CxxCH patterns) were analyzed with InterPro Scan to confirm multi-heme c-type cytochrome annotation (InterPro superfamily IPR036280). Phylogenetic analyses were conducted to test if the identified multi-heme cytochromes were closely related to proteins with known functions such as ammonia-forming nitrite reductase NrfA. The following selection of amino acid sequences was used for the tree calculation: (i) multi-heme cytochromes identified in the MAGs, (ii) 10 closest sequences from the Uniprot database, and (iii) members of the multi-heme cytochrome superfamily (IPR036280) with reviewed functional annotation. The sequences were aligned with MAFFT (e-ins-i method). A phylogenetic tree was calculated based on alignment sites conserved in ≥25% of the aligned sequences using FastTree [52] with the Le-Gascuel [53] substitution model.

The conductivity of pili was estimated based on *Pil*A protein sequences. Pili were considered potentially conductive if gaps between aromatic amino acids were shorter than 40 amino acids and percentages of aromatic amino acids exceeded 9%, as suggested by [54, 55]. Flavin-dependent extracellular electron transfer (FLEET) proteins of *Listeria monocytogenes*, as described by [56], were identified in Uniprot KB to obtain information on the COG orthologous groups and Pfam families these proteins are assigned to. Genes encoding proteins belonging to the same COG orthologous groups and exhibiting the same Pfam functional domains were searched in the MAGs to identify potential homologs of *Listeria* FLEET genes.

## Results

### Dissolved inorganic carbon and methane concentrations and methanogen abundances

**Figure 1:**
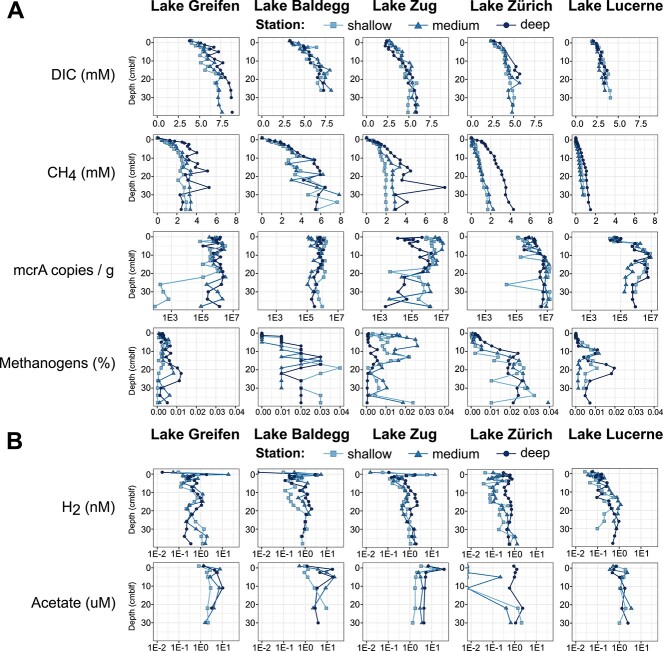
(A) Depth profiles of DIC and CH_4_ concentrations, absolute abundances of methanogens based on *mcr*A gene copy numbers, and relative abundances of methanogens ((*mcr*A copy numbers / total 16S rRNA copies) × 100). Notes: (i) fluctuations in CH_4_ concentrations in deeper layers of eutrophic lakes are presumably due to variations in outgassing intensity during sampling. (ii) The higher CH_4_ concentrations in eutrophic lakes were not reflected in higher *mcr*A copy numbers. (B) Depth profiles of methanogenic substrate concentrations (H_2_, acetate). DIC and CH_4_ concentrations were previously published in Fiskal et al. (2019) [6].

DIC and CH_4_ concentration trends reflect the stimulation of organic matter mineralization—in particular methanogenesis—rates in sediments of the eutrophic lakes, which receive higher organic matter inputs [6]. DIC concentration profiles follow typical concave-down trends, with steepest increases in surface sediments of eutrophic lakes, where organic matter mineralization rates are the highest (Fig. 1, 1st row). Thus, DIC concentration gradients increase from oligotrophic Lake Lucerne to the highly eutrophic Lake Baldegg and Lake Greifen. CH_4_ concentrations show similar trends in relation to trophic state (Fig. 1, 2nd row), and moreover indicate significant CH_4_ production all the way to the sediment surface in eutrophic lakes (also see [32]). Within most lakes, CH_4_ concentrations reached their highest values at the deepest station. This trend was not seen for DIC.

*McrA* copy numbers, used as a proxy for CH_4_-cycling archaeal abundance, were in the range of 10^4^–10^7^ copies g^−1^ wet sediment (Fig. 1, 3rd row). Copy numbers were generally higher in Lake Zurich and Lake Lucerne than the three eutrophic lakes, a trend that was also observed for total archaeal and bacterial 16S rRNA gene copies [31] and total cell counts [6]. This trend was opposite to the observed methanogenic activity increase with trophic state. Station-specific gene abundance profiles varied from near-constant abundances with depth (Lake Baldegg, deep and medium station in Lake Greifen) to increasing in the top ~10 cm and constant below (Lake Zurich), to decreasing with depth (shallow station in Lake Greifen, shallow and medium stations in Lake Zug). Some stations also had an increase in the top ~10 cm and decrease below (Lake Lucerne, deep station in Lake Zug).

We also calculated the contributions of CH_4_-cycling *Archaea* to total microbial populations based on ratios of *mcr*A to total 16S rRNA gene copy numbers from Han et al. (2020; Fig. 1, 4th row). Accordingly, contributions of CH_4_-cycling *archaea* to total microbial populations are very small (<0.04%) and show no clear trend in relation to trophic state.

### Concentrations of methanogenic energy substrates

Concentrations of the methanogenic substrates H_2_ and acetate (Fig. 1B) differ by three orders of magnitude (H_2_: mainly <2 nM; acetate: mostly 0.5 to 20 μM), but otherwise show several similarities. Both substrates are low in bottom water but have concentration peaks in the uppermost sediment layer (0–1 cm) of eutrophic lakes, which corresponds to the oxic-anoxic transition. These surface peaks are absent from the mesotrophic Lake Zurich and oligotrophic Lake Lucerne, where sedimentary concentrations of both substrates are lower (H_2_: mostly <1 nM; acetate: <4 μM). While both substrates show general concentration increases with depth in all lakes, there is a higher degree of subsurface variability in the three eutrophic lakes. Eutrophic lakes, moreover, display local peaks in H_2_ concentrations in the interval from 10–20 cm. Notably, sediments from this interval were deposited during the era of peak eutrophication, and remain highly enriched in OC content today [6].

### Stable carbon isotopic values of methane and dissolved inorganic carbon

**Figure 2:**
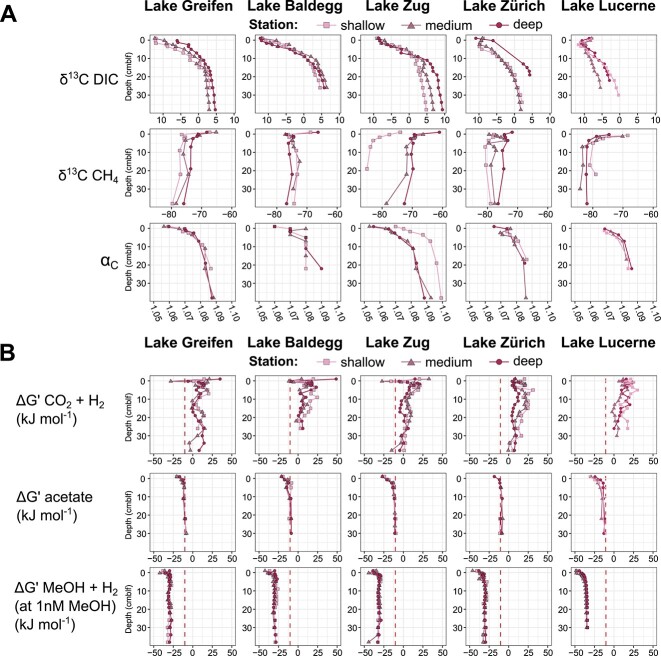
(A) Depth profiles of δ^13^C-CH_4_, δ^13^C-DIC, and ⍺_c_. (B) Gibbs energies (ΔG’ in kJ Mol^−1^) of methanogenesis reactions with different energy substrates. Red dashed lines indicate the biological energy quantum (−10 kJ Mol^−1^). For calculations involving methanol, we assumed an *in situ* methanol (MeOH) concentration of 1 nM. Note: Though the high ⍺_c_ values in (A) indicate methanogenesis via CO_2_ reduction as the dominant pathway, CO_2_ reduction with H_2_ was endergonic in most samples (B).

Carbon isotopic analyses of DIC and CH_4_ show similar trends across all lakes (Fig. 2, 1st and 2nd row). The δ^13^C-DIC values (range: −10 to +10 per mil) follow concave-down profiles in the top sediment, with the most negative values in surface sediment. The δ^13^C-CH_4_ values (range: −75 and − 95‰) show the opposite trend and decrease with depth. When the same depths are compared, δ^13^C-CH_4_ values are lower in Lake Zurich and Lake Lucerne than in the eutrophic lakes – a trend that was also seen in δ^13^C-DIC values and underscores the higher contribution of methanogenesis to organic matter mineralization rates in the eutrophic lakes [6]. The α_C_ values were generally >1.065 in sediments and increased with depth, showing no systematic trend in relation to trophic state (Fig. 1, 3rd row). Assuming mainly CH_4_ production from CO_2_ and acetate, these values indicate dominance of CO_2_ reduction.

### Thermodynamic calculations of methanogenesis

To examine the energetic potential of different methanogenesis reactions, we calculated the Gibbs energies of hydrogenotrophic and aceticlastic methanogenesis (Fig. 2B, 1st and 2nd row), as well as methanogenesis from methanol (methanol+H_2_: Fig. 2B, 3rd row; methanol only: Fig. S1). With exception of the sediment surface, ΔG’_CO2 + H2_ values were > 0 kJ mol^−1^, indicating that CO_2_ reduction with H_2_ was thermodynamically unfavorable. This result was surprising given that the δ^13^C-CH_4_ and α_C_-values (Fig. 2A) indicated predominantly methanogenesis by CO_2_ reduction, and that H_2_ is often assumed to be the main electron donor for methanogenic CO_2_ reduction in sediments. By contrast, aceticlastic and both methylotrophic reactions were exergonic. ΔG’_acetate_ values were slightly more exergonic than the theoretical minimum energy gain for energy conservation (biological energy quantum (BEQ); −10 kJ mol^−1^; [57, 58]) in surface sediment (0–5 cm) and stabilized at BEQ values below. The Gibbs energies of both methylotrophic reactions were even more exergonic (−50 to −30 kJ mol^−1^; assuming [methanol] = 1 nM) and in a similar range compared to each other, with on average slightly lower values for the reaction with methanol only. These highly similar Gibbs energy values of both methylotrophic reactions are independent of methanol concentrations. For instance, methanol concentrations of 1 μM would lower the Gibbs energies of both reactions by ~16 kJ mol^−1^.

### Community zonation of methane-cycling *Archaea*

**Figure 3:**
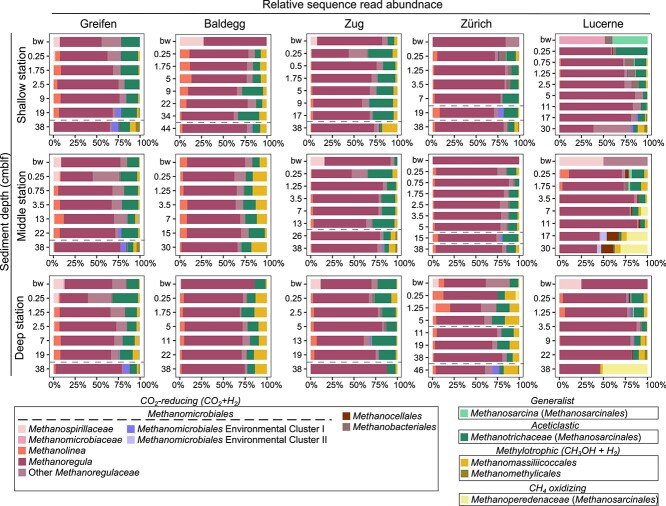
Community composition of CH_4_-cycling archaea based on *mcr*A gene amplicon sequences (bw = bottom water; sampled 5 to 10 cm above the sediment surface in each core). OTU-representative sequences were classified based on their placement in the phylogenetic tree of *mcr*A gene sequences (Fig. S2). Horizontal dashed lines in Lakes Greifen, Baldegg, and Zug indicate the timing of past shifts from oligotrophic to eutrophic, while in Lake Zurich they indicate changes from oligotrophic to eutrophic (lower line) and from eutrophic to mesotrophic (upper line).

*Mcr*A sequences were clustered into 1379 OTUs and taxonomically classified based on a phylogenetic tree (Fig. S2). Putative methanogens of the CO_2_-reducing order *Methanomicrobiales* made up the majority of *mcr*A sequences in all samples (Fig. 3; Fig. S3). Within the *Methanomicrobiales*, the genus *Methanoregula* accounted for 30 to 88% (average ± standard deviation (SD): 67 ± 12%) of total reads per sample. Other locally abundant *Methanomicrobiales* included the families *Methanospirillaceae* and *Methanomicrobiaceae*, the genus *Methanolinea*, and unclassified genus-level groups (*Methanolinea*-related, *Methanoregula*-related). Deeper layers in Lake Lucerne, and from the oligotrophic period or oligotrophic-eutrophic transition in Lake Greifen and Lake Zurich, harbored significant percentages of novel, family-level clusters of *Methanomicrobiales* (Environmental Clusters I and II). In addition, sequences of the CO_2_-reducing orders *Methanocellales* and *Methanobacteriales* were locally abundant in samples from Lake Lucerne.

Other groups included the aceticlastic genus *Methanothrix* (formerly *Methanosaeta*), which accounted for up to 36% (average ± SD: 12 ± 8%) of reads per sample (Fig. 3), and the methanol+H_2_-utilizing order *Methanomassiliicoccales* (up to 14% in Lake Baldegg; average ± SD: 5 ± 5%). The generalistic genus *Methanosarcina*, members of which can catabolize H_2_ + CO_2_, acetate, and methylated substrates, was also detected but rare, except in surface sediments and one bottom water sample from Lake Lucerne. The only methanotrophic *Archaea* detected belonged to the *Methanoperedenaceae*. This family was rare except in deep sediment layers of Lake Lucerne (up to 51%).

### Environmental factors shaping CH4-cycling archaeal communities

**Figure 4:**
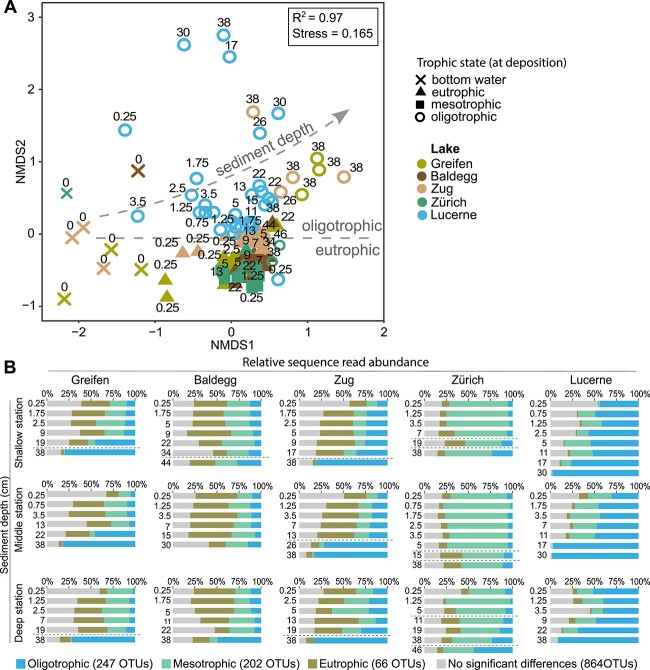
Impact of trophic status on CH_4_-cycling archaeal community composition based on *mcrA* gene amplicon sequences. (A) NMDS of samples based on Bray–Curtis dissimilarities at the OTU-level. Sample labels were reduced for legibility. Although no clear separation can be observed, most oligotrophic samples (circles) cluster separate from other trophic categories. Furthermore, a trend of ordering by increasing sediment depth, from left to right, can be observed for oligotrophic samples. (B) Cumulative read percentages of OTUs that showed significantly higher relative abundances in one of the three trophic categories by SIMPER (*P* < .05). The majority of reads belonged to OTUs that occurred at significantly higher percentages in one of the three trophic categories. Dashed horizontal lines in several plots indicate past changes in trophic state. In lakes Greifen, Baldegg, and Zug, these lines indicate past shifts from oligotrophic to eutrophic, while in Lake Zurich they indicate changes from oligo- to eutrophic (lower line) and eu- to mesotrophic (upper line).

﻿The diversity of CH_4_-cycling archaea was not significantly affected by the trophic status based on a comparison of species richness, alpha-diversity, or community evenness across lakes (Fig. S4). Similarly, a Non-linear Multidimensional Scaling (NMDS) of the samples based on their OTU-level community composition did not reveal a clear separation of samples based on trophic status. However, oligotrophic samples clustered toward one side of the plot and were more dispersed than the eutrophic samples, indicating higher community variation in sediments that were deposited under oligotrophic conditions (Fig. 4A).

To investigate whether certain taxa (OTUs) are more abundant in sediments deposited under a specific trophic state, we performed a SIMilarity PERcentage analysis (SIMPER; Fig. 4B). We identified 253 OTUs with a significantly higher average relative abundance in oligotrophic samples (12 to 97% of reads in any oligotrophic sample). The proportion of these “oligotrophic OTUs” increased with depth in Lake Lucerne and reached similarly high values in the deepest sediments from eutrophic lakes, which were deposited before these lakes became eutrophic. We also identified 68 and 205 OTUs with significantly higher percentage contributions in eutrophic (9 to 57%) and mesotrophic samples (33 to 74%), respectively. By comparison, most OTUs (852 out of the total of 1379) showed no significant differences in read percent contributions between trophic categories (1.7 to 67% of reads in any given sample).

### Methanogenesis pathways based on metagenome-assembled genomes (MAGs)

**Figure 5:**
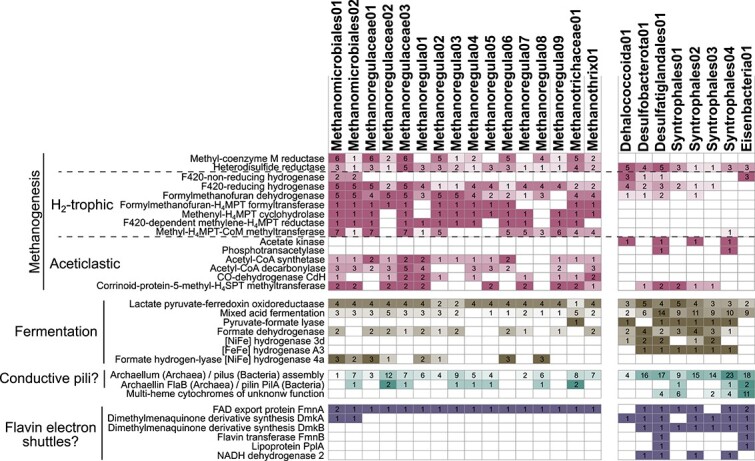
Genes indicative of methanogenesis, fermentation, and potential EET (rows) found within the MAGs of methanogens and potentially syntrophic microorganisms (columns). Numbers stand for differently annotated genes in a given category, e.g. encoding different subunits of an enzyme (e.g. methyl-CoM reductase), or different enzymes involved in a pathway (e.g. mixed-acid fermentation). For presence-absence of additional metabolic capabilities see Fig. S5; for the full list of annotations see Fig. S2.

The isotopic indications that methanogenesis is dominated by CO_2_ reduction (Fig. 2A) appear at odds with thermodynamic calculations, which indicate that CO_2_ reduction with H_2_, which is often considered the main electron source for CO_2_ reduction, is energetically unfavorable (Fig. 2B). To address this apparent contradiction, we analyzed genes indicative of methanogenesis pathways in 16 CH_4_-cycling archaeal MAGs completeness: >50%; genome contamination: <5%; Fig. 5, Fig. S5; Table S2) obtained from metagenomes of 35 sediment samples from the studied five lakes.

Based on phylogenomic analyses and complementary phylogenetic analyses of *mcr*A sequences, which were recovered from all but 3 MAGs (Fig. 5), 14 MAGs belonged to CO_2_-reducing *Methanomicrobiales* (12 *Methanoregulaceae*, 2 unassigned) and two to the aceticlastic family *Methanotrichaceae* (Fig. S5). At least four genes involved in hydrogenotrophic methanogenesis were present in all of these MAGs (Fig. 5). Consistent with past analyses [59, 60], these also included MAGs of *Methanotrichaceae*. Despite not using H_2_ as an electron source, members of this family express these genes during CO_2_-reduction via DIET [60]. All *Methanotrichaceae* MAGs also contained genes for aceticlastic methanogenesis via the high-affinity enzyme acetyl-CoA synthetase. Although all *Methanomicrobiales* MAGs also had an acetyl-CoA synthetase gene, the encoded enzyme belonged to a phylogenetically distinct group that is not involved in methanogenesis (Fig. S7). Consistent with the ability of certain *Methanomicrobiales* to oxidize formate to obtain H_2_ for CO_2_ reduction, eight MAGs included genes encoding H_2_-evolving [NiFe]-hydrogenase 4a and nine included genes encoding a formate dehydrogenase (6 MAGs encoded both). Genes indicative of methylotrophic methanogenesis were not detected.

In addition, we explored alternative pathways of CO_2_ reduction, e.g. via EET involving conductive structures (multi-heme cytochromes, conductive pilins). It should be noted here that although archaea are able to receive extracellular electrons, molecular mechanisms of EET on the archaeal side remain largely unknown and are therefore hard to identify via database-driven genome annotation. While no EET cytochromes were found, we identified genes encoding archaellum components in 14 MAGs and the gene encoding the archaellin monomer FlaB in eight MAGs. Archaellum of a hydrogenotrophic methanogen (Methanospirillum sp.) was recently shown to be electrically conductive [61].

### Potential syntrophic partner organisms of methanogenic *archaea*

We searched our metagenomes for potential partner organisms, that could supply electrons to methanogens by direct interspecies electron transfer (DIET), by identifying MAGs whose abundance was positively correlated to methanogenic MAGs (ρ > 0.75, *P* < .05; Table S3). A total of eight MAGs, all belonging to *Bacteria*, met these criteria, of which six belonged to the phylum *Desulfobacterota* (4 *Syntrophales*, 1 *Desulfatiglandales*, 1 SM23–61), one to *Chloroflexota* (GIF9 *Dehalococcoidia*), and one to *Eisenbacteria* (formerly *Latescibacterota*) (Fig. 5). Seven MAGs encoded genes for bifurcating and hydrogen evolving hydrogenases, i.e., ([NiFe] group 3d, [FeFe] group A3), and formate dehydrogenases, indicating both hydrogen- and formate-producing fermentation as possible catabolisms. In addition, indications for respiratory capabilities were found. Four MAGs (one of each *Desulfobacterota* SM32–61, *Desulfatiglandales*, *Dehalococcoidia*, and *Syntrophales*) also encoded genes for nitrate and sulfate reduction, whereas four others (three *Syntrophales,* one *Eisenbacteria*) had genes encoding NADH:ubiquinone oxidoreductase (respiratory complex I) subunits (Fig. S6).

Within these MAGs, we found several genes encoding membrane-bound multi-heme cytochromes with 3–8 heme binding sites (Fig. 5). All MAGs, moreover, contained genes involved in pilus assembly, and three had *pil*A genes encoding the monomer constituting the pilus itself. Besides *pil*A sequences, we identified homologs of genes that are believed to be involved in FLEET in *Listeria monocystogenes* [56] in the MAGs of potential syntrophs. The presence of these genes in bacterial MAGs that positively correlate in abundance with methanogenic MAGs confirms the potential for these bacteria to engage in DIET with methanogenic partners.

## Discussion

Increases in the supply of algal organic matter to lake sediments due to eutrophication have been shown to increase sedimentary CH_4_ production and atmospheric CH_4_ emissions from lakes [6, 62–64]. Less is known about how these increases in OC supply and methanogenesis rates affect sedimentary communities of methanogens. Here we use time–resolved sedimentary records of five temperate lakes in central Switzerland to examine how changes in trophic state over the last century have affected the abundance, community structure, and metabolic pathways of methanogens.

### Eutrophication impacts on abundance of methanogenic *Archaea*

We show that, despite increased sedimentary input and burial of algal OC in the three eutrophic lakes [31, 65], which coincides with elevated sedimentary CH_4_ concentrations and methanogenesis rates [6], the population size of CH_4_-cycling *Archaea* was higher in the meso- and oligotrophic lakes (Fig. 1). This trend is opposite to that previously documented in two lakes on the Yunnan plateau, China [64]. However, in the lakes studied, the same trend was also observed for total cell numbers [6], total 16S rRNA gene copy numbers [30, 31], and copy numbers of genes of aerobic methanotrophy [32], all of which were lower in eutrophic lake sediments. The fact that the eutrophic lake sediments have overall lower microbial population densities than oligo- and mesotrophic sediments with lower OC inputs and metabolic activities has been explained with higher grazing pressure by deposit-feeding oligochaetes [6], which occur at high densities (~1000 to 9000 specimen m^−2^) in the upper 10 to 20 cm of sediments in these lakes [30]. Yet, differences in microbial population size do not necessarily translate into differences in activity, as cell-specific metabolic rates can vary by orders of magnitude among microorganisms in response to energy availability [57]. Recent research has, moreover, demonstrated that even under intense deposit-feeding with constant microbial biomass removal by macrofauna, total microbial activity can exceed that in sediments where these macrofauna are absent [66]. We thus propose that the elevated input of labile organic matter in the three eutrophic lakes supports significantly higher cell-specific and total methanogenesis rates, despite (grazing-induced) smaller populations of microorganisms, including methanogens.

### Impacts of trophic state on methanogenic community structure

Independent of the trophic state at the time of sediment deposition, *mcr*A amplicon and metagenome analyses indicate that all samples were dominated by CO_2_-reducing methanogens (83% ± 9%), with the majority of reads belonging to the genus *Methanoregula* and other closely related *Methanoregulaceae* (order *Methanomicrobiales*; Fig. 3). Although the type species *Methanoregula boonei* was originally isolated from an acidic oligotrophic peat bog [67], other uncultured members of *Methanoregulaceae* have since been shown to be widespread in lake sediments [22, 68, 69]. Phylogenetic (Fig. S2B) and phylogenomic analyses (Fig. S5) show that the *Methanoregula* in our samples belong to previously unsequenced species. Other widespread, putatively CO_2_-reducing *Methanomicrobiales* include members of *Methanospirillaceae* (mainly bottom water and surface sediment), *Methanolineaceae* (no clear pattern in distribution), and five uncultivated environmental clusters (Fig. S2B). Among the latter, the two dominant environmental clusters (clusters I and II) are closely related to *Methanoregulaceae* (Fig. S2B). Notably, these two clusters are rare in sediments deposited under eutrophic conditions and are the only putatively CO_2_-reducing taxa with an apparent trophic preference.

Aceticlastic *Methanosaetaceae* (*Methanotrichaceae*) and methylotrophic methanogenic taxa (methanol+H_2_; *Methanomassiliicoccales*) were also widespread, but consistently less abundant than CO_2_ reducers and show no noticeable trends in relation to present or past trophic state (Fig. 3). Among the aceticlastic methanogens, the obligately aceticlastic *Methanotrichaceae* dominated over facultatively aceticlastic *Methanosarcina* consistent with previous studies on lake sediments [70, 71] and on the physiologies of both groups [72, 73]. The latter have shown that *Methanothrix* outcompete *Methanosarcina* at acetate concentrations in the low micromolar range, as in the sediments studied (Fig. 1B). This is due to the higher affinity to acetate of acetyl-CoA synthetase, an enzyme that activates acetate in *Methanothrix*, compared to acetate kinase, which is used by *Methanosarcina* [72, 73]. Matching these studies, MAGs of *Methanotrichaceae* in our study also contained genes encoding high-affinity acetyl-CoA synthetases (Fig. 5).

As in other recent studies on lake sediments [22, 74], methylotrophic methanogens consisted largely of *Methanomassiliicoccales* (class *Thermoplasmata*), which reduce methanol to CH_4_ using H_2_. The presence of significant percentages of *Methanomassiliicoccales* in almost all samples, and at much lower percentages the equally hydrogenotrophic methanol-reducing *Methanomethylicales* (*Verstraetearchaeota*; Fig. 3), indicates that methanol is the main methylotrophic methanogenic substrate in the lakes studied. Noticeably absent were potentially competing, methanol-disproportionating genera of *Methanosarcinaceae* (e.g. *Methanococcoides*, *Methanolobus*, *Methanomethylovorans*), which are widespread in marine sediments [68, 75]. Our calculations indicate methylotrophic reactions with and without H_2_ to have similar Gibbs energy yields per mol methanol in the lakes studied (Fig. 2, Fig. S1; also see Results). Thus, factors other than *in situ* energy yields, e.g. differences in substrate affinity of essential enzymes, presence of syntrophic partner organisms, or availability of essential metal catalysts and cofactors, may explain the observed dominance of methanol-reducing over methanol-disproportionating methanogens.

Different from higher taxonomic levels, methanogen communities at the OTU-level show clear structuring in relation to the trophic state at the time of sediment deposition (Fig. 4). This pattern is similar to that previously observed for CH_4_-oxidizing bacteria in the same samples [32] and indicates that sediments of the five lakes are dominated by closely-related but nonetheless distinct species (or subspecies). We propose that, despite having the same basic ecological function, slight differences in ecophysiologies select for distinct methanogenic species assemblages in sediments that were deposited under different trophic regimes.

### What drives the contributions of aceticlastic and methylotrophic methanogenesis?

Aceticlastic methanogenesis has long been considered the dominant methanogenic pathway in freshwater systems [20, 21, 26]. Yet, a growing number of studies, including this study, is showing that this trend is not universal, and that methanogenesis by CO_2_ reduction dominates in certain lakes [76, 77].

The reasons for the uniform dominance of CO_2_ reduction over other methanogenic pathways in the lakes studied here remain elusive (also see next section), as does the fact that relative abundances of aceticlastic and methylotrophic methanogens do not follow documented trends. For instance, while some studies have proposed that higher algal input of fresh organic matter favors aceticlastic methanogenesis [11, 26, 78], we do not observe an increase in the contribution of aceticlastic methanogens (Fig. 3) or decrease in α_C_-values in surface sediments of eutrophic lakes (Fig. 2A), where algal biomass inputs and contributions would be the highest. Similarly, even though methanol is produced during the breakdown of vascular plant polymers, e.g. xylan, pectin, and lignin [24, 25, 79], the percentage of methylotrophic methanogens does not increase in sediments of Lakes Zug and Lucerne, which have elevated contributions of terrestrial plant-derived organic matter [31].

In the case of acetate, it is possible that only small amounts of labile organic matter reach the methanogenesis zone. Sedimentation rates at the stations in the three eutrophic lakes are in the range of 0.22 to 0.37 cm yr^−1^ [6] and macrofaunal sediment mixing (reworking) was shown to be minimal both in the field [33] and laboratory [30], despite high abundances of deeply burrowing tubificids. In the absence of vertical mixing, organic matter will have undergone months of aerobic degradation, including depletion of its most reactive fractions, by the time it reaches the anoxic zone at 1–2 mm sediment depth. In the case of methylotrophic methanogenesis, the reason could be related to the fact that it is now known that pectin and lignin are not only produced by vascular plants, but also by microalgae [75, 80]. Fermentative breakdown of phytoplankton biomass could thus be an additional methanol source and explain why percentages of methylotrophic methanogens do not follow terrestrial contributions of organic matter.

While the above arguments offer reasons for the absence of aceticlastic or methylotrophic pathway trends in relation to trophic state or organic matter source, they per se do not explain why neither are quantitatively more important. One explanation is that the relative contributions of both pathways is controlled upstream by fermentative organisms. If only a minor portion of fermentation reactions yields acetate as an end product in the lakes studied, then this would explain why acetate is not the dominant methanogenic substrate. Aceticlastic methanogens (*Methanothrix*) may still control *in situ* acetate concentrations, driving these concentrations down until the apparent thermodynamic energy threshold for the reaction is reached (−10 kJ mol^−1^; Fig. 2B). Yet, this does not require acetate to be a dominant fermentation product or methanogenic substrate. In addition, competing anaerobic acetate oxidizing microorganisms may divert acetate away from aceticlastic methanogens. Indeed, thermodynamic calculations indicate that in all lakes *in situ* energy yields of anaerobic acetate oxidation are higher (~0 to 10 cm sediment depth) or similar (below ~10 cm) to those of aceticlastic methanogenesis (Fig. S8). In the case of methanol, the high energy yield of methanogenesis from methanol+H_2_ suggests that methylotrophic methanogens, unlike aceticlastic methanogens, do not reach minimum energy thresholds. For these methanogens, substrate availability may be the main limiting factor. This would match past observations that methanol is not a major fermentation product in anoxic sediments [81–83].

### What is the electron source for methanogenic carbon dioxide reduction?

Lastly, and most importantly, why do C-isotopic compositions (Fig. 2A) and methanogenic community structure indicate dominance of CO_2_ reduction, while Gibbs energies of hydrogenotrophic CO_2_ reduction are largely endergonic? Potential explanations include the presence of chemical microenvironments [84, 85] or involvement of electron sources other than H_2_. Neither scenario can be ruled out, however, the latter appears more likely.

If chemical microenvironments are the explanation, then, according to our calculations, H_2_ concentrations within these microenvironments would need to be 10- to 50-fold higher than measured H_2_ concentrations in bulk sediments for Gibbs energies to reach the minimum energy threshold of −10 kJ per mol of CH_4_ produced. Labile organic particles that support locally high rates of H_2_ production by fermentation could generate such H_2_ concentration peaks. Yet, it is unclear how these concentration peaks would be maintained in the face of rapid diffusive equilibration with much lower H_2_ concentrations in surrounding sediments. Immediate efficient scavenging of produced H_2_ can only decrease the H_2_ concentration down to the “*minimum biologically useful level”* resulting in a Gibbs energy near −10 kJ per mol [58], while bulk H_2_ concentrations measured in the studied sediments are far below this level. Moreover, most of our samples were from buried subsurface layers that have experienced decades of organic matter degradation and are unlikely to be rich in labile organic particles. More generally, while microniches can explain methanogenesis in environments where methanogenesis is a background process, e.g. oxic water columns or sulfate-reducing marine surface sediments [85, 86], it is difficult to envision how they would be the main sites of CH_4_ production in sediments where methanogenesis is the dominant respiration reaction [6].

By the same reasoning, CO_2_ reduction involving formate as an electron source does not provide a plausible explanation either. This process relies on the intracellular conversion of formate to CO_2_ and H_2_, after which the resulting H_2_ is used to reduce CO_2_ to CH_4_ [87]. However, microbial cells cannot contain H_2_, which—unless enzymatically metabolized—diffuses rapidly through microbial cell walls to the surrounding sediment [88]. Taking into account the additionally very low cell-specific catabolic activity of subsurface microorganisms in the sediments studied [6], it seems unlikely that methanogens maintain intracellular H_2_ concentrations that are 10- to 50-fold higher than those in surrounding sediments. Moreover, previous multi-day incubation experiments involving formate addition (100 μM) to anoxic sediments from Lake Lucerne resulted in no significant stimulation of methanogenesis [89].

A more plausible explanation than microniches or diffusive electron carriers is CO_2_ reduction by direct interspecies electron transfer (DIET), e.g., between methanogens and syntrophic partners via conductive protein structures. Culture-based studies have shown the ability of certain bacteria that are canonically known as secondary fermenters (e.g., *Syntrophus aciditrophicus*) to also perform DIET [55]. In addition, aceticlastic and hydrogenotrophic methanogens can grow electrotrophically in cultures [30, 110–112]. DIET between *Archaea* and *Bacteria* is known to be mediated by bacterial conductive structures (e.g. pili, cytochromes) [60, 90]. In addition, conductive structures or direct contact can mediate EET through environmental substances, including iron oxides [91], iron–sulfur minerals [92], and humic substances [93].

Support for DIET comes from the positive correlations between relative abundances of methanogenic MAGs and MAGs of certain anaerobic bacteria, as well as the genetic repertoire of these bacterial MAGs. The majority of the latter were classified as *Deltaproteobacteriota*, of which most belong to uncultured families of *Syntrophales*. Cultured representatives of the genus *Syntrophus* are known to form syntrophic associations with methanogens in co-cultures and anaerobic digesters (e.g. [94–96]) and can produce conductive pili [55]. It has even been suggested that members of this genus prefer DIET over H_2_ or formate exchange [55]. While Walker et al. [55] suggested that *S. aciditrophicus* relies solely on conductive pili for DIET and does not encode multi-heme cytochromes, we found c-type cytochromes with three to eight heme domains encoded in our *Syntrophales* MAGs and in the published genome of *S. aciditrophicus* ([97]; also see Supplementary material). Additionally, we found pilus assembly genes in all potentially syntrophic bacterial MAGs, although—perhaps due to MAG incompleteness—several were missing the gene for the pilin monomer. The pilin genes we found in our MAGs only partially fulfill the conductivity criteria [55], i.e. while gaps between aromatic amino acids are sufficiently short the proportion of aromatic amino acids is too low to safely conclude conductivity. Yet, pilin genes of *Syntrophales* that fulfill both criteria were found in the unbinned fraction of the metagenome. Lastly, we found genes homologous to the ones involved in EET mediated by extracellular flavin shuttles [56] in MAGs of all potential syntrophs.

Identifying extracellular electron uptake genes in the archaeal MAGs is more challenging due to the lack of molecular understanding and genetic markers for DIET or EET in Archaea [27, 98]. Most Archaea do not have multi-heme cytochromes and only one conductive archaellin has been described so far [61]. While we detected archaellin genes in most of our MAGs, the conductivity of these genes remains unclear.

## Conclusions

Our combination of isotopic, community compositional, and genomic approaches indicates that methanogenesis by CO_2_ reduction is the dominant methanogenic pathway in five temperate lakes irrespective of past or present trophic state, organic matter inputs, or electron acceptor distributions. At the species- or subspecies-level, the methanogenic community appears structured in relation to lake trophic state at the time of sediment deposition; however, this structuring is not evident at the genus-level and above. Similarly, the methanogenic community is not organized in relation to electron acceptor distributions. Our findings contrast with previous studies on total microbial community structure [34] and methane-oxidizing bacterial community structure [36] within sediments of the same lakes, both of which showed clear zonations in relation to trophic history and electron acceptor distributions. A potential explanation is that, despite major differences in past and present environmental conditions, anaerobic organic matter decomposition in these lakes produces a small set of products at ratios that are similar and consistently favor CO_2_ reduction over other methanogenic pathways. This explanation is at odds with past research that concluded dominance of aceticlastic methanogenesis in freshwater sediments [21]. To add further oddity, thermodynamic calculations rule out a predominance of H_2_-dependent CO_2_ reduction. Based on genomic analyses and environmental correlations of CO_2_-reducing methanogens with syntrophic bacteria that are known to engage in EET, we propose that CO_2_ reduction via DIET is the dominant methanogenic pathway in the lakes studied and a widespread process in sedimentary environments. Identifying the mechanisms by which electrons are transferred *in situ*, e.g. directly from partner organisms or via electrically charged chemical structures, is a priority for future research.

## Supplementary Material

5lakes_Supplementary_Information_final_ycae089

File_S1_Function_search_m-gens_syntrophs_ycae089

File_S2_Lakes_methanogens_and_syntrophs_annotations_ycae089

## Data Availability

All *mcrA* amplicon sequences can be accessed under BioProject PRJNA1066858 on the National Center for Biotechnology Information (NCBI) website. The 16S rRNA gene amplicon reads have been published previously [31] and are available under project number PRJNA577818 on the same website. Samples used for metagenomic analysis can be found under the ID Gs0142423 on JGI GOLD (https://gold.jgi.doe.gov). Metagenomic datasets are publicly available at the JGI genome portal (https://genome.jgi.doe.gov/portal/) under the proposal ID 504756 and at NCBI/ENA (PRJNA620348–PRJNA620356, PRJNA620360–PRJNA620364, PRJNA654670–PRJNA654680). Metagenome-assembled genomes of methanogens and potential syntrophs as well as functional annotation tables can be accessed on Figshare (DOI: http://10.6084/m9.figshare.24421363).

## References

[1] Mendonça R , Müller RA, Clow D et al. Organic carbon burial in global lakes and reservoirs. Nat Commu*n* 2017;8:1694. 10.1038/s41467-017-01789-6 29162815 PMC5698497

[2] Dean WE , Gorham E. Magnitude and significance of carbon burial in lakes, reservoirs, and peatlands. Geolog*y* 1998;26:535–8. 10.1130/0091-7613(1998)026<0535:MASOCB.3.CO;2

[3] Hedderich R , Whitman WB. Physiology and biochemistry of the methane-producing Archaea. The Prokaryote*s* 2006;2:1050–79.

[4] Bastviken D , Cole J, Pace M et al. Methane emissions from lakes: dependence of lake characteristics, two regional assessments, and a global estimate. Glob Biogeochem Cycle*s* 2004;18:GB4009. 10.1029/2004GB002238

[5] Dean JF , Middelburg JJ, Röckmann T et al. Methane feedbacks to the global climate system in a warmer world. Rev Geophy*s* 2018;56:207–50. 10.1002/2017RG000559

[6] Fiskal A , Deng L, Michel A et al. Effects of eutrophication on sedimentary organic carbon cycling in five temperate lakes. Biogeoscience*s* 2019;16:3725–46. 10.5194/bg-16-3725-2019

[7] Zhu Y , Purdy KJ, Eyice Ö et al. Disproportionate increase in freshwater methane emissions induced by experimental warming. Nat Clim Chan*g* 2020;10:685–90. 10.1038/s41558-020-0824-y

[8] Weber T , Wiseman NA, Kock A. Global ocean methane emissions dominated by shallow coastal waters. Nat Commu*n* 2019;10:4584. 10.1038/s41467-019-12541-7 31594924 PMC6783430

[9] Whitman WB , Bowen TL, Boone DR. The methanogenic bacteria. In: Rosenberg E., EF D.L., Lory S. et al. (eds.), The Prokaryotes: Other Major Lineages of Bacteria and the Archaea. Berlin, Heidelberg: Springer Berlin Heidelberg, 2014, 123–63.

[10] Lovley DR , Goodwin S. Hydrogen concentrations as an indicator of the predominant terminal electron-accepting reactions in aquatic sediments. Geochim Cosmochim Act*a* 1988;52:2993–3003. 10.1016/0016-7037(88)90163-9

[11] Conrad R . Importance of hydrogenotrophic, aceticlastic and methylotrophic methanogenesis for methane production in terrestrial, aquatic and other anoxic environments: a mini review. Pedospher*e* 2020;30:25–39. 10.1016/S1002-0160(18)60052-9

[12] Dridi B , Fardeau M-L, Ollivier B et al. *Methanomassiliicoccus luminyensis* gen. nov., sp. nov., a methanogenic archaeon isolated from human faeces. Int J Syst Evol Microbio*l* 2012;62:1902–7. 10.1099/ijs.0.033712-0 22859731

[13] Borrel G , O’Toole PW, Harris HMB et al. Phylogenomic data support a seventh order of methylotrophic methanogens and provide insights into the evolution of methanogenesis. Genome Biol Evo*l* 2013;5:1769–80. 10.1093/gbe/evt128 23985970 PMC3814188

[14] Sorokin DY , Merkel AY, Abbas B et al. *Methanonatronarchaeum thermophilum* gen. nov., sp. nov. and ‘Candidatus Methanohalarchaeum thermophilum’, extremely halo(natrono)philic methyl-reducing methanogens from hypersaline lakes comprising a new euryarchaeal class Methanonatronarchaeia classis nov. Int J Syst Evol Microbio*l* 2018;68:2199–208. 10.1099/ijsem.0.002810 29781801 PMC6978985

[15] Nobu MK , Narihiro T, Kuroda K et al. Chasing the elusive Euryarchaeota class WSA2: genomes reveal a uniquely fastidious methyl-reducing methanogen. ISME *J* 2016;10:2478–87. 10.1038/ismej.2016.33 26943620 PMC5030686

[16] Vanwonterghem I , Evans PN, Parks DH et al. Methylotrophic methanogenesis discovered in the archaeal phylum Verstraetearchaeota. Nat Microbio*l* 2016;1:16170. 10.1038/nmicrobiol.2016.170 27694807

[17] Evans PN , Boyd JA, Leu AO et al. An evolving view of methane metabolism in the Archaea. Nat Rev Microbio*l* 2019;17:219–32. 10.1038/s41579-018-0136-7 30664670

[18] Lever MA , Teske AP. Diversity of methane-cycling archaea in hydrothermal sediment investigated by general and group-specific PCR primers. Appl Environ Microbio*l* 2015;81:1426–41. 10.1128/AEM.03588-14 25527539 PMC4309701

[19] Mayumi D , Mochimaru H, Tamaki H et al. Methane production from coal by a single methanogen. Scienc*e* 2016;354:222–5. 10.1126/science.aaf8821 27738170

[20] Conrad R . Contribution of hydrogen to methane production and control of hydrogen concentrations in methanogenic soils and sediments. FEMS Microbiol Eco*l* 1999;28:193–202. 10.1111/j.1574-6941.1999.tb00575.x

[21] Whiticar MJ , Faber E, Schoell M. Biogenic methane formation in marine and freshwater environments: CO2 reduction vs. acetate fermentation—isotope evidence. Geochim Cosmochim Act*a* 1986;50:693–709. 10.1016/0016-7037(86)90346-7

[22] Bertolet BL , West WE, Armitage DW et al. Organic matter supply and bacterial community composition predict methanogenesis rates in temperate lake sediments. Limnol Oceanogr Let*t* 2019;4:164–72. 10.1002/lol2.10114

[23] Lyautey E , Billard E, Tissot N et al. Seasonal dynamics of abundance, structure, and diversity of methanogens and methanotrophs in lake sediments. Microb Eco*l* 2021;82:559–71. 10.1007/s00248-021-01689-9 33538855

[24] Donnelly MI , Dagley S. Production of methanol from aromatic acids by pseudomonas putida. J Bacterio*l* 1980;142:916–24. 10.1128/jb.142.3.916-924.1980 7380811 PMC294117

[25] Schink B , Zeikus JG. Microbial methanol formation: a major end product of pectin metabolism. Curr Microbio*l* 1980;4:387–9. 10.1007/BF02605383

[26] Whiticar MJ . Carbon and hydrogen isotope systematics of bacterial formation and oxidation of methane. Chem Geo*l* 1999;161:291–314. 10.1016/S0009-2541(99)00092-3

[27] Holmes DE , Rotaru A-E, Ueki T et al. Electron and proton flux for carbon dioxide reduction in Methanosarcina barkeri during direct interspecies electron transfer. Front Microbio*l* 2018;9:3109. 10.3389/fmicb.2018.03109 30631315 PMC6315138

[28] Rotaru A-E , Posth NR, Löscher CR et al. Interspecies interactions mediated by conductive minerals in the sediments of the iron rich meromictic Lake La Cruz, Spain. Limnetica 2019;38:21–40.

[29] Zhang J , Lu Y. Conductive Fe3O4 nanoparticles accelerate syntrophic methane production from butyrate oxidation in two different lake sediments. Front Microbio*l* 7:1316. 10.3389/fmicb.2016.01316 PMC4992681 27597850

[30] Fiskal A , Anthamatten E, Deng L et al. Carbon sources of benthic fauna in temperate lakes across multiple trophic states. Biogeoscience*s* 2021;18:4369–88. 10.5194/bg-18-4369-2021

[31] Han X , Schubert CJ, Fiskal A et al. Eutrophication as a driver of microbial community structure in lake sediments. Environ Microbio*l* 2020;15115:1462–2920. 10.1111/1462-2920.15115 32510812

[32] van Grinsven S , Meier DV, Michel A et al. Redox zone and trophic state as drivers of methane-oxidizing bacterial abundance and community structure in lake sediments. Front Environ Sc*i* 2022;10:857358. 10.3389/fenvs.2022.857358

[33] van Dijk J , Fernandez A, Müller IA et al. Oxygen isotope fractionation in the siderite-water system between 8.5 and 62 °C. Geochim Cosmochim Act*a* 2018;220:535–51. 10.1016/j.gca.2017.10.009

[34] Conrad R . Quantification of methanogenic pathways using stable carbon isotopic signatures: a review and a proposal. Org Geoche*m* 2005;36:739–52. 10.1016/j.orggeochem.2004.09.006

[35] Hoehler TM , Alperin MJ, Albert DB et al. Thermodynamic control on hydrogen concentrations in anoxic sediments. Geochim Cosmochim Act*a* 1998;62:1745–56. 10.1016/S0016-7037(98)00106-9

[36] Wiesenburg DA , Guinasso NL Jr. Equilibrium solubilities of methane, carbon monoxide, and hydrogen in water and sea water. J Chem Eng Dat*a* 1979;24:356–60. 10.1021/je60083a006

[37] Glombitza C , Pedersen J, Røy H et al. Direct analysis of volatile fatty acids in marine sediment porewater by two-dimensional ion chromatography-mass spectrometry. Limnol Oceanogr Method*s* 2014;12:455–68. 10.4319/lom.2014.12.455

[38] Stumm W , Morgan JJ. Aquatic Chemistry: Chemical Equilibria and Rates in Natural Waters. John Wiley & Sons, Inc., New York 1996.

[39] Millero F . The activity coefficients of non-electrolytes in seawater. Mar Che*m* 2000;70:5–22. 10.1016/S0304-4203(00)00011-6

[40] Zhuang G-C , Heuer VB, Lazar CS et al. Relative importance of methylotrophic methanogenesis in sediments of the Western Mediterranean Sea. Geochim Cosmochim Act*a* 2018;224:171–86. 10.1016/j.gca.2017.12.024

[41] Lever MA , Torti A, Eickenbusch P et al. A modular method for the extraction of DNA and RNA, and the separation of DNA pools from diverse environmental sample types. Front Microbio*l* 2015;6:476. 10.3389/fmicb.2015.00476 PMC4436928 26042110

[42] Magoč T , Salzberg SL. FLASH: fast length adjustment of short reads to improve genome assemblies. Bioinformatic*s* 2011;27:2957–63. 10.1093/bioinformatics/btr507 21903629 PMC3198573

[43] Edgar RC . Search and clustering orders of magnitude faster than BLAST. Bioinformatic*s* 2010;26:2460–1. 10.1093/bioinformatics/btq461 20709691

[44] Schmieder R , Edwards R. Quality control and preprocessing of metagenomic datasets. Bioinformatic*s* 2011;27:863–4. 10.1093/bioinformatics/btr026 21278185 PMC3051327

[45] Edgar RC . UNOISE2: improved error-correction for Illumina 16S and ITS amplicon sequencing. bioRxi*v* 2016;081257:476.

[46] Lever MA , Alperin MJ, Hinrichs K-U et al. Zonation of the active methane-cycling community in deep subsurface sediments of the Peru trench. Front Microbio*l* 2023;14:1192029. 10.3389/fmicb.2023.1192029 37250063 PMC10213550

[47] Oksanen J , Simpson G, Blanchet F et al. Vegan: community ecology package. R package version 2.6-4. http://cran.r-project.org/package=vegan.

[48] Wickham H . ggplot2: Elegant Graphics for Data Analysis. New York: Springer, 2016.

[49] Chaumeil P-A , Mussig AJ, Hugenholtz P et al. GTDB-Tk: a toolkit to classify genomes with the genome taxonomy database. Bioinformatic*s* 2019;36:1925–7. 10.1093/bioinformatics/btz848 31730192 PMC7703759

[50] Dombrowski N , Williams TA, Sun J et al. Undinarchaeota illuminate DPANN phylogeny and the impact of gene transfer on archaeal evolution. Nat Commu*n* 2020;11:3939. 10.1038/s41467-020-17408-w 32770105 PMC7414124

[51] Lloréns-Rico V , Vieira-Silva S, Gonçalves PJ et al. Benchmarking microbiome transformations favors experimental quantitative approaches to address compositionality and sampling depth biases. Nat Commu*n* 2021;12:3562. 10.1038/s41467-021-23821-6 34117246 PMC8196019

[52] Price MN , Dehal PS, Arkin AP. FastTree 2—approximately maximum-likelihood trees for large alignments. PLoS On*e* 2010;5:e9490. 10.1371/journal.pone.0009490 20224823 PMC2835736

[53] Le SQ , Gascuel O. An improved general amino acid replacement matrix. Mol Biol Evo*l* 2008;25:1307–20. 10.1093/molbev/msn067 18367465

[54] Walker DJ , Adhikari RY, Holmes DE et al. Electrically conductive pili from pilin genes of phylogenetically diverse microorganisms. ISME *J* 2018;12:48–58. 10.1038/ismej.2017.141 28872631 PMC5739001

[55] Walker DJF , Nevin KP, Holmes DE et al. *Syntrophus* conductive pili demonstrate that common hydrogen-donating syntrophs can have a direct electron transfer option. ISME *J* 2020;14:837–46. 10.1038/s41396-019-0575-9 31896792 PMC7031330

[56] Light SH , Su L, Rivera-Lugo R et al. A flavin-based extracellular electron transfer mechanism in diverse Gram-positive bacteria. Natur*e* 2018;562:140–4. 10.1038/s41586-018-0498-z 30209391 PMC6221200

[57] Lever MA , Rogers KL, Lloyd KG et al. Life under extreme energy limitation: a synthesis of laboratory- and field-based investigations. FEMS Microbiol Re*v* 2015;39:688–728. 10.1093/femsre/fuv020 25994609

[58] Hoehler TM , Alperin MJ, Albert DB et al. Apparent minimum free energy requirements for methanogenic Archaea and sulfate-reducing bacteria in an anoxic marine sediment. FEMS Microbiol Eco*l* 2001;38:33–41. 10.1111/j.1574-6941.2001.tb00879.x

[59] Smith KS , Ingram-Smith C. *Methanosaeta*, the forgotten methanogen? Trends Microbio*l* 2007;15:150–5. 10.1016/j.tim.2007.02.002 17320399

[60] Rotaru A-E , Shrestha PM, Liu F et al. A new model for electron flow during anaerobic digestion: direct interspecies electron transfer to *Methanosaeta* for the reduction of carbon dioxide to methane. Energy Environ Sc*i* 2013;7:408–15.

[61] Walker DJF , Martz E, Holmes DE et al. The archaellum of *Methanospirillum hungatei* is electrically conductive. MBi*o* 2019;10:10. 10.1128/mBio.00579-19 PMC6469973 30992355

[62] West WE , Coloso JJ, Jones SE. Effects of algal and terrestrial carbon on methane production rates and methanogen community structure in a temperate lake sediment. Freshw Bio*l* 2012;57:949–55. 10.1111/j.1365-2427.2012.02755.x

[63] Beaulieu JJ , DelSontro T, Downing JA. Eutrophication will increase methane emissions from lakes and impoundments during the 21st century. Nat Commu*n* 2019;10:1375. 10.1038/s41467-019-09100-5 30914638 PMC6435651

[64] Yang Y , Chen J, Tong T et al. Influences of eutrophication on methanogenesis pathways and methanogenic microbial community structures in freshwater lakes. Environ Pollu*t* 2020;260:114106. 10.1016/j.envpol.2020.114106 32041086

[65] Han X , Tolu J, Deng L et al. Long-term preservation of biomolecules in lake sediments: potential importance of physical shielding by recalcitrant cell walls. PNAS Nexu*s* 2022;1:gac076. 10.1093/pnasnexus/pgac076 PMC9896894 36741427

[66] Deng L , Fiskal A, Bölsterli D et al. Differential impact of two major polychaete guilds on microbial communities in marine sediments: a microcosm study. Front Mar Sc*i* 2023;10:720. 10.3389/fmars.2023.1119331

[67] Bräuer SL , Cadillo-Quiroz H, Ward RJ et al. *Methanoregula boonei* gen. nov., sp. nov., an acidiphilic methanogen isolated from an acidic peat bog. Int J Syst Evol Microbio*l* 2011;61:45–52. 10.1099/ijs.0.021782-0 20154331

[68] Wen X , Yang S, Horn F et al. Global biogeographic analysis of methanogenic archaea identifies community-shaping environmental factors of natural environments. Front Microbio*l* 2017;8:1339. 10.3389/fmicb.2017.01339 28769904 PMC5513909

[69] Borrel G , Jézéquel D, Biderre-Petit C et al. Production and consumption of methane in freshwater lake ecosystems. Res Microbio*l* 2011;162:832–47. 10.1016/j.resmic.2011.06.004 21704700

[70] Chan OC , Claus P, Casper P et al. Vertical distribution of structure and function of the methanogenic archaeal community in Lake Dagow sediment. Environ Microbio*l* 2005;7:1139–49. 10.1111/j.1462-2920.2005.00790.x 16011751

[71] Conrad R , Klose M, Claus P et al. Methanogenic pathway, ^13^C isotope fractionation, and archaeal community composition in the sediment of two clear-water lakes of Amazonia. Limnol Oceanog*r* 2010;55:689–702.

[72] Jetten MSM , Stams AJM, Zehnder AJB. Methanogenesis from acetate: a comparison of the acetate metabolism in *Methanothrix soehngenii* and *Methanosarcina* spp. FEMS Microbiol Re*v* 1992;88:181–98. 10.1111/j.1574-6968.1992.tb04987.x

[73] Berger S , Welte C, Deppenmeier U. Acetate activation in *Methanosaeta thermophila*: characterization of the key enzymes pyrophosphatase and acetyl-CoA synthetase. Archae*a* 2012;2012:1–10. 10.1155/2012/315153 PMC3426162 22927778

[74] Yakimovich KM , Orland C, Emilson EJS et al. Lake characteristics influence how methanogens in littoral sediments respond to terrestrial litter inputs. ISME *J* 2020;14:2153–63. 10.1038/s41396-020-0680-9 32424248 PMC7367837

[75] Fischer PQ , Sánchez-Andrea I, Stams AJM et al. Anaerobic microbial methanol conversion in marine sediments. Environ Microbio*l* 2021;23:1348–62. 10.1111/1462-2920.15434 33587796 PMC8048578

[76] Conrad R , Claus P, Casper P. Characterization of stable isotope fractionation during methane production in the sediment of a eutrophic lake, lake Dagow, Germany. Limnol Oceanog 2009;54:457–71.

[77] Yang Y , Li N, Wang W et al. Vertical profiles of sediment methanogenic potential and communities in two plateau freshwater lakes. Biogeoscience*s* 2017;14:341–51. 10.5194/bg-14-341-2017

[78] Schwarz JIK , Eckert W, Conrad R. Response of the methanogenic microbial community of a profundal lake sediment (Lake Kinneret, Israel) to algal deposition. Limnol Oceanog*r* 2008;53:113–21. 10.4319/lo.2008.53.1.0113

[79] Rosell K-G , Svensson S. Studies of the distribution of the 4-O-methyl-d-glucuronic acid residues in birch xylan. Carbohydr Re*s* 1975;42:297–304. 10.1016/S0008-6215(00)84271-8

[80] Sista Kameshwar AK , Qin W. Structural and functional properties of pectin and lignin–carbohydrate complexes de-esterases: a review. Bioresour Bioproces*s* 2018;5:1–16.

[81] Schink B , Zeikus JG. Microbial ecology of pectin decomposition in anoxic lake sediments. Microbiolog*y* 1982;128:393–404. 10.1099/00221287-128-2-393

[82] Lovley DR , Klug MJ. Methanogenesis from methanol and methylamines and acetogenesis from hydrogen and carbon dioxide in the sediments of a eutrophic lake. Appl Environ Microbio*l* 1983;45:1310–5. 10.1128/aem.45.4.1310-1315.1983 16346271 PMC242456

[83] Conrad R , Claus P. Contribution of methanol to the production of methane and its ^13^C-isotopic signature in anoxic rice field soil. Biogeochemistr*y* 2005;73:381–93. 10.1007/s10533-004-0366-9

[84] Jørgensen BB . Bacterial sulfate reduction within reduced microniches of oxidized marine sediments. Mar Bio*l* 1977;41:7–17. 10.1007/BF00390576

[85] Anderson JG , Meadows PS. Microenvironments in marine sediments. Proc Royal Soc Edinburgh *B* 1978;76:1–16.

[86] Bartosiewicz M , Venetz J, Läubli S et al. Detritus-hosted methanogenesis sustains the methane paradox in an alpine lake. Limnol Oceanog*r* 2023;68:248–64. 10.1002/lno.12263

[87] Sparling R , Daniels L. Source of carbon and hydrogen in methane produced from formate by *Methanococcus thermolithotrophicus*. J Bacterio*l* 1986;168:1402–7. 10.1128/jb.168.3.1402-1407.1986 3782041 PMC213652

[88] Finke N , Hoehler TM, Jørgensen BB. Hydrogen ‘leakage’ during methanogenesis from methanol and methylamine: implications for anaerobic carbon degradation pathways in aquatic sediments. Environ Microbio*l* 2007;9:1060–71. 10.1111/j.1462-2920.2007.01248.x 17359276

[89] Eickenbusch P . Microbial Cycling of Formate and Other Low-Molecular Weight Aliphatic Organic Acids in Anoxic Environments. ETH Zürich: Ph.D, 2019.

[90] Rotaru A-E , Shrestha PM, Liu F et al. Direct interspecies electron transfer between *Geobacter metallireducens* and *Methanosarcina barkeri*. Appl Environ Microbio*l* 2014;80:4599–605. 10.1128/AEM.00895-14 24837373 PMC4148795

[91] Kato S , Hashimoto K, Watanabe K. Methanogenesis facilitated by electric syntrophy via (semi)conductive iron-oxide minerals. Environ Microbio*l* 2012;14:1646–54. 10.1111/j.1462-2920.2011.02611.x 22004041

[92] Kato S , Igarashi K. Enhancement of methanogenesis by electric syntrophy with biogenic iron-sulfide minerals. Microbiology Ope*n* 2019;8:e00647. 10.1002/mbo3.647 29877051 PMC6436484

[93] Lovley DR , Coates JD, Blunt-Harris EL et al. Humic substances as electron acceptors for microbial respiration. Natur*e* 1996;382:445–8. 10.1038/382445a0

[94] Jackson BE , Bhupathiraju VK, Tanner RS et al. *Syntrophus aciditrophicus* sp. nov., a new anaerobic bacterium that degrades fatty acids and benzoate in syntrophic association with hydrogen-using microorganisms. Arch Microbio*l* 1999;171:107–14. 10.1007/s002030050685 9914307

[95] Sieber JR , Le HM, McInerney MJ. The importance of hydrogen and formate transfer for syntrophic fatty, aromatic and alicyclic metabolism. Environ Microbio*l* 2014;16:177–88. 10.1111/1462-2920.12269 24387041

[96] Galushko A , Kuever J. Syntrophaceae. In: Bergey’s Manual of Systematics of Archaea and Bacteria. John Wiley & Sons, Inc., New York, 2020, 1–3.

[97] McInerney MJ , Rohlin L, Mouttaki H et al. The genome of *Syntrophus aciditrophicus*: life at the thermodynamic limit of microbial growth. Proc Natl Acad Sci US*A* 2007;104:7600–5. 17442750 10.1073/pnas.0610456104 PMC1863511

[98] Gao K , Lu Y. Putative extracellular electron transfer in methanogenic archaea. Front Microbio*l* 2021;12:611739. 10.3389/fmicb.2021.611739 33828536 PMC8019784

