# Correction to: Hydrogen–independent CO_2_ reduction dominates methanogenesis in five temperate lakes that differ in trophic states

**DOI:** 10.1093/ismeco/ycaf082

**Published:** 2025-05-27

This is a correction to: Dimitri Meier, Sigrid van Grinsven, Anja Michel, Philip Eickenbusch, Clemens Glombitza, Xingguo Han, Annika Fiskal, Stefano Bernasconi, Carsten J Schubert, Mark A Lever, Hydrogen–independent CO_2_ reduction dominates methanogenesis in five temperate lakes that differ in trophic states, *ISME Communications*, Volume 4, Issue 1, January 2024, ycae089, https://doi.org/10.1093/ismeco/ycae089

In the ‘Results’ section the sentence:

Accordingly, contributions of CH_4_-cycling *archaea* to total microbial populations are very small (<0.04%) and show no clear trend in relation to trophic state.

has been corrected to read:

Accordingly, contributions of CH_4_-cycling *archaea* to total microbial populations are small (mostly <1%) and show no clear trend in relation to trophic state.

Additionally, in Figure 1, the *y*-axis label ‘Methanogens (%)’ has been corrected to read ‘mcrA copies / total 16S copies’.

