# Supplementary material for: Hydrogen–independent CO2 reduction dominates methanogenesis in five temperate lakes that differ in trophic states
Source: ISME Commun. 2024 Jun 21;4(1):ycae089. doi: 10.1093/ismeco/ycae089 (PMC11235125; doi:10.1093/ismeco/ycae089)
Supplement: 5lakes_Supplementary_Information_final_ycae089 [file 5lakes_supplementary_information_final_ycae089.pdf]

Supplementary Information

Hydrogen-independent CO_2 reduction dominates methanogenesis in five temperate lakes that differ in trophic states

Dimitri Meier*, Sigrid van Grinsven, Anja Michel, Philip Eickenbusch, Clemens Glombitza, Xingguo Han, Annika Fiskal, Stefano Bernasconi, Carsten J. Schubert, Mark A. Lever*

Supplementary Methods

Additional information on H_2 quantification method

The quantification of H_2 concentrations in anoxic sediments using the incubation method requires that samples remain fully anoxic throughout the incubation period, and that equilibrium H_2 concentrations between sediment and overlying N_2 headspace are established prior to H_2 measurement. To ensure both prerequisites were met, we performed the following precautions.

O_2 contamination was kept to a minimum by only using previously unpierced butyl septa. Moreover, immediately after sampling using cut-off syringes, sediments were transferred to sterile 20-mL headspace incubation vials, that were – to remove any O_2 - instantly flushed for ≥1 minute using N_2 prior to sealing with septa and crimping. Lastly, since butyl rubber septa have previously been shown to contain significant concentrations of O_2, which can then leak into anoxic incubation vials and cause significant O_2 contamination [41], we ensured that all butyl septa were anoxic prior to incubation. To remove O_2, we pre-incubated autoclaved butyl septa in gas-tight glass containers under an N_2 atmosphere in the presence of O_2-consuming AnaeroGen bags for a duration of at least 5 days prior to sampling. We also checked for H_2 release from septa after AnaeroGen treatment and found this to be insignificant.

To ensure that equilibrium H_2 concentrations between sediment and headspace were established prior to H_2 measurement, we performed preliminary tests at elevated temporal sampling resolution (0h, 5h, 10h, 24h, 48h). Using anoxic sediments from 4-6 and 24-28 cm sediment depth at the deep station in Lake Lucerne, we were able to demonstrate that after rapid initial increases, H_2 concentrations had stabilized after 10 h, and remaining stable throughout the remainder of the tests. Despite removing 2 mL of headspace gas from each 20-mL vial for each measurement, and replacing this 2 mL with N_2 gas, the headspace [H_2] remained stable. This further, confirmed the establishment and subsequent maintenance of equilibrium H_2 concentrations between sediment and the overlying headspace.

Metagenomic sequence analysis

Extracted DNA was shipped to the Joint Genome Institute (JGI), Walnut Creek, CA, USA, where it was sequenced and assembled according to the standard JGI pipeline. Briefly the raw Illumina reads were corrected using BBtools [1] program bbcms v. 38.34 with the following command line options: *mincount=2 highcountfraction=0.6*. The error-corrected readsets were assembled using metaSPAdes assembler v. 3.13.0. [2] without error-correction step, since the reads were error-corrected before, and with iteration through k-mer sizes of *33, 55, 77, 99, 127*. The assembled sequences were annotated with IMGAP annotation pipeline (v. 5.0.11) [3]. Genes were identified using a combination of following gene callers: CRT v.1.8.2, GeneMark.hmm-2 v.1.05, INFERNAL v.1.1.2, Prodigal v.2.6.3, tRNAscan-SE v.2.0.5 and functional annotations using by lastal v.983 and HMMER v.3.1b2 using following reference databases: Rfam v.13.0, IMG-NR (07-06.2019); SMART (01.06.2016); COG (2003 version), TIGRFAM v.15.0, SuperFamily v1.75, Pfam v.30, Cath-Funfam v.4.2.0. Additionally a separate annotation of carbohydrate-active enzymes was performed using dbCAN v. 2.0.11 [4] and the CAZY database [5]. Decaheme cytochromes potentially involved in extracellular electron transfer were annotated with FeGenie v. 1.2 [6] and searched manually based on heme-site detection (For details see *Metagenome function search and analysis*).

Metagenome binning

Each of the 25 metagenome assemblies annotated by the JGI were subjected to differential-coverage and tetranucleotide-frequencies based binning. For this purpose, error-corrected reads from 25 metagenomes sequenced by the JGI and additional 10 metagenomes sequenced at the FGCZ were mapped to each of the 25 annotated assemblies to collect coverage information. Reads were mapped with bbmap v. 38.49 and an identity cut-off of 95%. Each assembly was binned into putative population genomes by MetaBAT v. 2.12.1 [7], MaxBin v.2.2.4 [8] and CONCOCT v. 0.4.1 [9] and summarized into a non-redundant set of bins by DASTool v. 1.1.0 [10] with a score threshold of 0.2. Metagenomic bins from all 25 metagenomes were analyzed for their size, completeness, redundancy, and contig length with CheckM v.1.1.3 [11] and dereplicated with dRep v.1.4.3 [12] keeping the bin with the best metrics for each set of replicate genomes stemming from different samples. The functional annotations of this dereplicated set of 986 bins were combined into one file (large table stored as an SQL database). The bins were further manually refined using Anvi'O v. 7 [13] in order to reduce "contamination", i.e. duplications of genes expected to appear as single-copy in a given microbial lineage. Contigs were removed from the bins based on divergent coverage profiles and tetranucleotide frequencies visualized in Anvi'O. After the refinement, the completeness and duplication of the bins were evaluated with CheckM once again and only bins with a completeness over 50% and contamination below 5% were considered for further analysis (434 bins).

The MAGs were taxonomically classified by GTDB-Tk [14] placing them into the Genome Taxonomy Database phylogenetic trees based on the concatenated alignment of protein sequences of 120

bacterial [15] and 53 archaeal [16] phylogenetic markers. Based on tree topology, relative evolutionary distance, and average nucleotide identity, GTDB-Tk classifies genomes as belonging to a certain taxonomic group or representing a distinct novel clade. For the MAGs belonging to the class *Methanomicrobia*, the classification was confirmed by calculating a de-novo phylogenetic tree based on amino-acid positions conserved in at least 25% of the sequences of concatenated alignments of 53 phylogenetic marker proteins [16]. Tree was calculated with FastTree v. 2.1.11 [17] utilizing the Le-Gascuel [18] substitution model, starting with a Bio-Neighbour-Joining tree [19], and rescaling the branch length to optimize Gamma20 likelihood.

Metagenome function search and analysis

Genes encoding functions indicative of specific metabolic pathways were searched in the combined annotation table of dereplicated genomic bins by means of SQL queries based on IMG-assigned gene product names, EC numbers, COG, and Pfam annotations. Carbohydrate degrading enzymes were searched based on CAZY database annotations. Criteria used for search of the reported genes can be found in File S1 (A log of SQL queries). Amino acid sequences of hydrogenase large subunits were uploaded to HydDB [20] for classification of the exact hydrogenase type.

Proteins containing multiple heme domains that escaped FeGenie annotation were identified by searching for the CxxCH (cysteine, 2 x any amino acid, cysteine, histidine) patterns in the amino acid sequences indicating a heme-binding site. The proteins were analyzed with InterPro Scan [21] to confirm that they were multi-heme c-type cytochromes (InterPro superfamily IPR036280). Phylogenetic analyses were conducted to test if the identified multi-heme cytochromes were closely related to proteins with known functions such as ammonia-forming nitrite reductase *Nrf*A. Amino acid sequences of the following selection of genes were used for the tree calculation: i) multi-heme cytochromes identified in the MAGs, ii) 10 closest sequences of i) from the Uniprot database, and iii) members of the multi-heme cytochrome superfamily (IPR036280) with reviewed functional annotation. The sequences were aligned with MAFFT (e-ins-i method) [22]. The phylogenetic tree was calculated based on sites that were conserved in ≥25% of the aligned sequences using FastTree with BioNJ starting tree, Le-Gascuel substitution model, and optimization of gamma likelihood.

Potential conductivity of pili was estimated based on protein sequences encoded by *pil*A genes by determining the percentage of aromatic amino acids and the length of gaps between them. Pili were considered potentially conductive if the gaps between aromatic amino acids were shorter than 40 amino acids and the percentages of aromatic amino acids were higher than 9% based on *Geobacter* and *Syntrophus* pilin analysis by Walker *et al*. [23, 24].

Proteins involved in flavin-dependent extracellular electron transfer (FLEET) are yet not well described and their annotation in genomes other than relatives of Listeria might be fuzzy. Therefore, FLEET proteins of *Listeria monocytogenes*, as described by [25] were identified in Uniprot knowledge base in order to obtain information on the COG orthologous groups they are assigned to

and Pfam functional domains of these proteins. Genes encoding proteins belonging to the same COG orthologous groups and exhibiting same Pfam functional domains were searched in the MAGs to identify potential homologues of the Listeria FLEET genes.

Supplementary Results and Discussion

Multi-heme cytochrome and conductive pilin genes identification in MAGs

The majority of the potentially syntrophic MAGs was classified as *Deltaproteobacteriota*, of which most belong to uncultured families of *Syntrophales*. Cultured representatives of the genus *Syntrophus* form syntrophic associations with methanogens in cultures and anaerobic digesters (e.g. [26–28]). Moreover, the pili of *Syntrophus aciditrophicus* were recently shown to be conductive and it was suggested that DIET might be its preferred mode of syntrophic interaction over hydrogen or formate exchange [24].

Investigating this possibility based on 35 environmental metagenomes, we found genes that could be indicative of EET in the MAGs of potentially syntrophic species. These include genes for multi-heme cytochromes of yet unknown functions and genes encoding potentially conductive pili. While [24] suggested that *Syntrophus aciditrophicus* does not encode multi-heme cytochromes, we were able to find several c-type cytochromes with three to eight heme domains encoded in our *Syntrophales* MAGs, as well as a published genome of *Syntrophus aciditrophicus* [29]. These genes escaped the annotation by the Fe-genie tool used by Walker et al. [24] and were only detected by manual searches for sequences with multiple heme binding motifs. In other cases these sequences were annotated as nitrite reductase, a multi-heme cytochrome protein itself.

The presence of a nitrite reductase gene in the genome of *Syntrophus aciditrophicus*, which is not known to be capable of denitrification [26], was reported previously [29]. In this context, it is worth noting that nitrite reductase is closely related to other multi-heme cytochromes [30] and a weak nitrite reducing activity of iron-reducing multi-heme cytochromes was observed in e.g. the thermophilic iron-reducer *Thermincola potens [31]*. Hence *Syntrophales* might use multi-heme cytochromes for EET after all, in addition to conductive pili. We have found pilus assembly genes in all potentially syntrophic bacterial MAGs. However, the majority of the MAGs with genes for pilus assembly are missing the gene for the pilin monomer itself, most likely due to MAG incompleteness. According to criteria suggested by [24] the pilin genes found in our MAGs fulfill the criterion of short gaps between aromatic amino acids to enable electron hopping, but do not fulfill the criterion of total proportion of aromatic amino acids in the protein. Pilin genes fulfilling the sequence-based conductivity criteria and assigned to *Syntrophales* were found in the unbinned fraction of the metagenome. High conservation of the sequences might have led to several *Syntrophales* populations sharing almost identical pilin sequences, which has led to difficulties of assigning the DNA sequence to a specific

MAG. Additionally, we found genes homologous to the ones involved in EET mediated by extracellular flavin shuttles in Gram-positive anaerobic fermenter *Listeria monocytogenes* [25] in the MAGs of potential syntrophs.

Identifying potential extracellular electron uptake gene in the archaeal MAGs is even more challenging due to the lack of good molecular understanding and genetic markers [32, 33]. Most Archaea do not have multi-heme cytochromes, which are used by bacteria to take up electrons from an extracellular surface. Furthermore, *Methanosarcina barkeri* has been shown to not require such cytochromes for EET [34]. As for conductive pili, only one conductive archaellin has been described so far and the criteria for determining conductivity are not yet well established [35]. Just as in case with bacterial pili, the archaellin genes found in our MAGs fulfill the short gaps between aromatic amino acids, but not the total aromatic amino acid proportion criterion.

Supplementary Tables and Figures

Table S1: Thermodynamic constants of aqueous educts and products under standard conditions. ΔG_f°, ΔH_f°, and ΔV_f° refer to standard Gibbs energies, enthalpies, and molal volumes of formation, respectively.

**Compound**	**ΔG_f° (kJ mol^{-1})**	**ΔH_f° (kJ mol^{-1})**	**ΔV_f° (cm^3 mol^{-1})**	**Source**
proton (H^+)	0.0	0.0	0.0	[36]
hydrogen (H_2) (aq)	17.6	-4.2	25.2	[37, 38]
water	-237.2	-285.8	18.0	[39]
bicarbonate	-586.9	-692.0	24.6	[36]
formate	-351.0	-425.7	26.2	[38]
acetate	-369.4	-486.4	40.5	[38]
methanol	-175.39	-246.48	38.17	[38]
methane	-34.47	-87.96	37.30	[38]

Table S2: Metagenome-assembled genome metrics. 'L50' values indicate assembly quality. Half of the sequence information is on contigs longer than the L50 length. 'Completeness' is the percentage of genes present in all other genomes of the lineage present in the MAG. 'Contamination' indicates the percentage of lineage-specific single-copy genes present in multiple, divergent copies. 'Strain heterogeneity' refers to the percentage of lineage-specific single-copy genes present in multiple, yet highly similar copies (> 90% amino acid identity). 'ANI' refers to average nucleotide identity, whereas 'RED' is the relative evolutionary distance [15].

MAG name	Sample with the best assembly	Tool to produce best bin	MAG size (Mbp)	# of contigs	L50 (bp)	GC content (%)	Complete-ness (%)	Contami-nation (%)	Strain hetero-geneity (%)	Taxonomic classification GTDB-Tk	Accession of closest genome	ANI to closest genome (%)	Fraction of genomes aligned (%)	RED value
Methanomicrobiales01	Greifen, 38 cm	CONCOCT	1.72	438	5199	64	92	0.0	2.64	Unclassified Methanomicrobiales				0.69
Methanomicrobiales02	Zug, 38 cm	MetaBAT2	1.13	224	5334	66	74	0.7	0.00	Unclassified Methanomicrobiales				0.61
Methanoregulaceae01	Zürich, 0.25 cm	MetaBAT2	2.35	191	16953	53	97	0.5	1.09	Methanomicrobiales; unclassified Methanoregulaceae				0.77
Methanoregulaceae02	Zug, 13 cm	MetaBAT2	0.72	185	3854	57	53	0.0	0.00	Methanomicrobiales; unclassified Methanoregulaceae				0.81
Methanoregulaceae03	Greifen, 0.25 cm	MetaBAT2	2.89	131	41343	50	99	0.3	0.34	Methanomicrobiales; Methanoregulaceae; CAIKOD01 sp.	GCA_903828975.1	87	72	0.98
Methanoregula09	Lucerne, 38 cm	MetaBAT2	1.76	295	6513	50	84	0.4	0.89	Methanomicrobiales; Methanoregulaceae; Methanoregula sp.	GCA_003141335.1	89	70	0.97
Methanoregula04	Zug, 0.25 cm	MetaBAT2	1.37	275	5447	49	82	1.0	1.01	Methanomicrobiales; Methanoregulaceae; Methanoregula sp.	GCA_003141335.1	85	77	0.97
Methanoregula06	Zürich, 0.25 cm	MaxBin	1.22	619	2056	52	66	1.6	1.65	Methanomicrobiales; Methanoregulaceae; Methanoregula sp.				0.96
Methanoregula08	Baldegg, 38 cm	MetaBAT2	1.04	237	4427	50	63	0.0	0.65	Methanomicrobiales; Methanoregulaceae; Methanoregula sp.	GCA_003141335.1	90	80	0.97
Methanoregula02	Baldegg, 5 cm	MaxBin	1.01	622	1628	50	62	3.2	10.77	Methanomicrobiales; Methanoregulaceae; Methanoregula sp.	GCA_003141335.1	86	89	0.95
Methanoregula03	Zürich, 5 cm	MetaBAT2	0.70	179	3951	50	61	0.0	0.19	Methanomicrobiales; Methanoregulaceae; Methanoregula sp.	GCA_002495055.1	83	80	0.95
Methanoregula05	Zug, 38 cm	MetaBAT2	1.26	299	4402	48	55	4.0	0.44	Methanomicrobiales; Methanoregulaceae; Methanoregula sp.	GCA_003141335.1	84	66	0.96
Methanoregula07	Zug, 38 cm	MetaBAT2	1.06	252	4129	51	51	0.0	0.00	Methanomicrobiales; Methanoregulaceae; Methanoregula sp.	GCA_003141335.1	90	72	0.97
Methanoregula01	Baldegg, 0.25 cm	MaxBin	0.98	597	1614	50	48	4.6	8.13	Methanomicrobiales; Methanoregulaceae; Methanoregula sp.	GCA_003141335.1	86	79	0.95
Methanotrichaceae01	Zug, 13 cm	MaxBin	2.63	1151	2596	49	64	2.9	1.72	Methanotrichales; unclassified Methanotrichaceae (previously Methanisaetaceae)				0.83
Methanothrix01	Zug, 5 cm	CONCOCT	1.22	722	1743	55	58	3.3	2.22	Methanotrichales; Methanotrichaceae; Methanothrix (previously Methanisaeta)				0.92
Dehalococcoida01	Zug, 13 cm	MetaBAT2	1.28	257	5072	54	65	0.0	0.99	Chloroflexota; Dehalococcoidia; GIF9; UBA5629; CAIMUM01 sp.	GCA_903844685.1	85	70	0.98
Desulfatiglandales01	Baldegg, 11 cm	MetaBAT2	3.78	633	7062	53	84	1.3	2.58	Desulfobacterota; Desulfobacteria; Desulfatiglandales; HGW-15; DSXZ01 sp.	GCA_011367735.1	80	52	0.93
Desulfobacterota01	Baldegg, 5 cm	MaxBIn	3.91	1807	2376	55	84	3.8	2.27	Desulfobacterota; unclassified SM23-61				0.73
Syntrophales01	Zug, 0.25 cm	MetaBAT2	1.78	302	6674	54	73	1.3	1.94	Desulfobacterota; Syntrophia; Syntrophales; unclassified Fen-1087	GCA_003161855.1	79	19	0.85
Syntrophales02	Zug, 38 cm	MetaBAT2	2.14	347	7177	48	89	1.9	1.94	Desulfobacterota; Syntrophia; Syntrophales; UBA2185; UBA2185 sp.	GCA_003508565.1	82	47	0.93
Syntrophales03	Baldegg, 15 cm	MetaBAT2	1.85	372	5361	48	78	1.2	0.39	Desulfobacterota; Syntrophia; Syntrophales; UBA2185; UBA2185 sp.	GCA_003508565.1	82	53	0.93
Syntrophales04	Zug, 13 cm	MetaBAT2	2.16	370	6482	56	77	3.9	1.47	Desulfobacterota; Syntrophia; Syntrophales; UBA4778 sp.				0.77
Eisenbacteria01	Greifen, 7 cm	MetaBAT2	2.69	476	5996	72	71	0.0	0.00	Eisenbacteria; RBG-16-71-46; unclassified RBG-16-71-46 (previously Latescibacterota)				0.61

	Methanoregula08	Methanoregula03	Methanoregula04	Methanoregula02	Methanoregula01	Methanoregulaceae02
Methanoregula03	0.35	NA	0.83	0.96	0.87	0.47
Methanoregula04	0.19	0.83	NA	0.87	0.76	0.51
Methanoregula02	0.35	0.96	0.87	NA	0.94	0.53
Methanoregula01	0.33	0.87	0.76	0.94	NA	0.48
Dehalococcoida01	0.18	0.71	0.81	0.76	0.68	0.58
Desulfatiglandales01	0.24	0.73	0.64	0.79	0.80	0.49
Desulfobacterota01	0.25	0.72	0.63	0.79	0.80	0.42
Syntrophales01	-0.05	0.52	0.78	0.59	0.51	0.29
Syntrophales03	0.05	0.55	0.67	0.72	0.81	0.36
Syntrophales02	0.26	0.78	0.92	0.82	0.69	0.58
Syntrophales04	0.39	0.52	0.68	0.59	0.45	0.82
Eisenbacteria01	0.20	0.69	0.57	0.77	0.76	0.52

Table S3: MAGs with a Spearman's correlation coefficient of $\rho>0.75$ and $p<0.05$ to at least one methanogen MAG. The red box marks correlations of methanogenic MAGs with MAGs of potential bacterial syntrophs. All six methanogenic MAGs that passed the threshold of >0.5x genome coverage and presence in >20% of the samples belonged to putatively CO_2-reducing *Methanoregulaceae*.

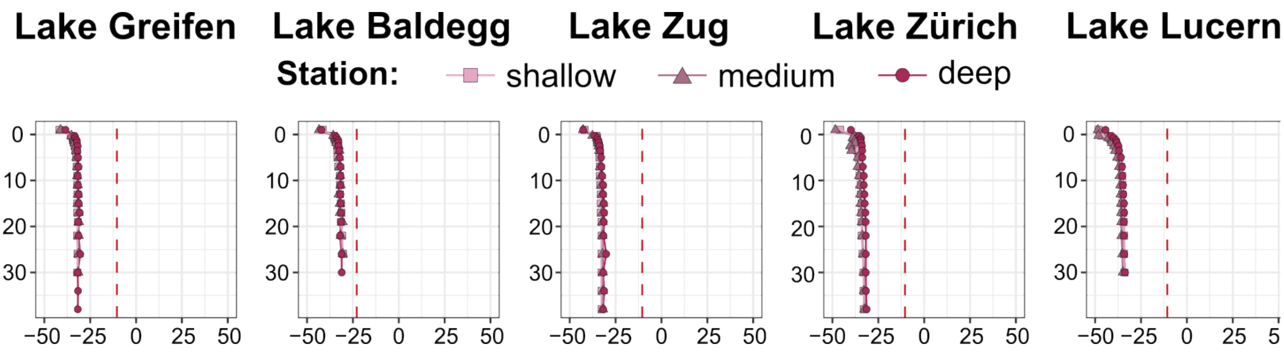

Figure S1: Gibbs energies (ΔG*r*'; in kJ mol^{-1}) of methylotrophic methanogenesis reactions involving methanol as the energy substrate, and assuming an *in situ* methanol concentration of 1 nM. Red dashed lines indicate the biological energy quantum (-10 kJ mol^{-1}). Note: depth profiles of calculated Gibbs energies were nearly identical for all three stations per lake.

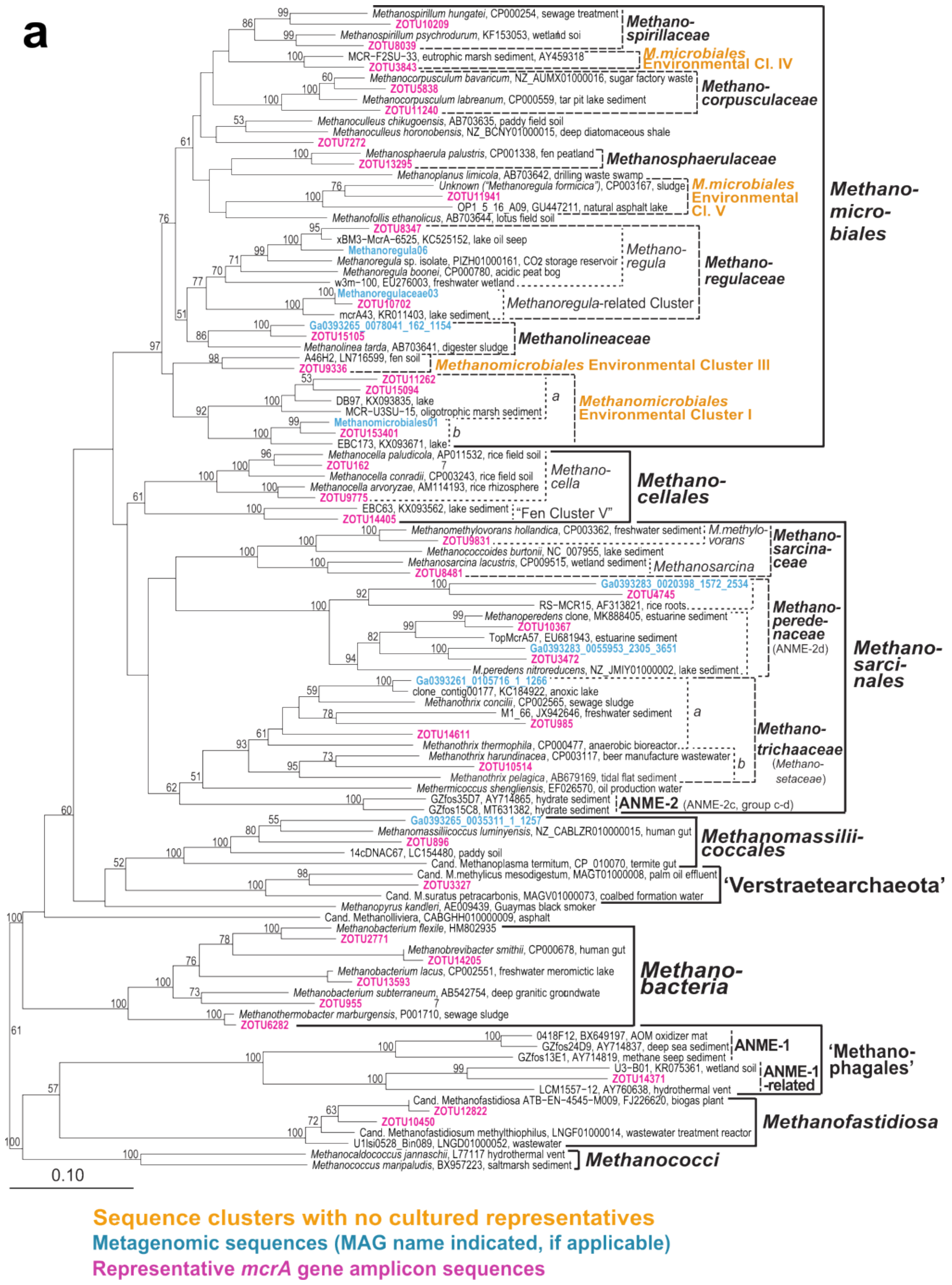

Figure S2: (a) Neighbour-joining phylogenetic tree of all detected *mcrA* gene clusters. On following page: **(b)** Neighbour-joining phylogenetic tree of *mcrA* gene sequences of *Methanomicrobiales*. To ensure legibility, only representative OTU and metagenomic sequences are shown.

b

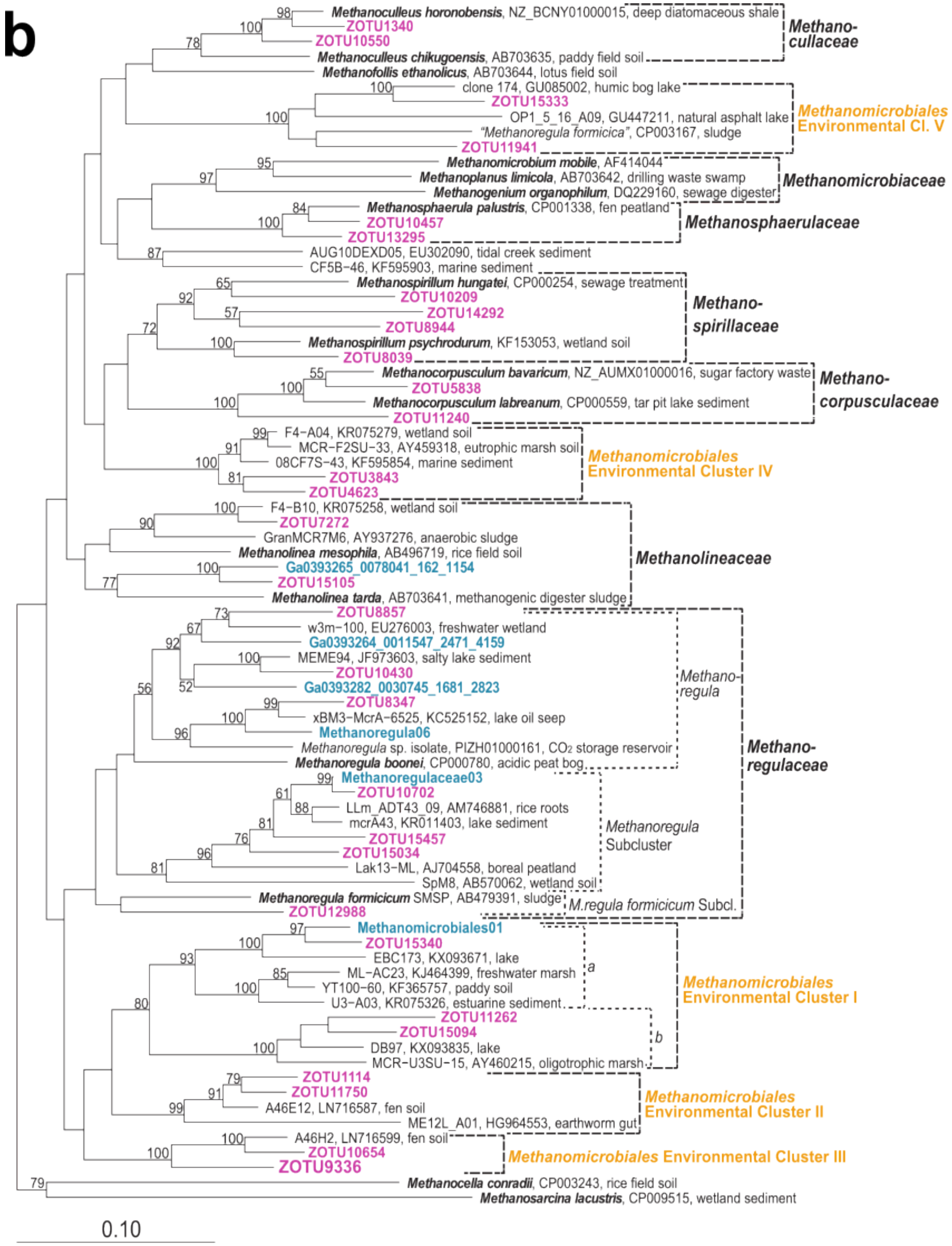

Sequence clusters with no cultured representatives
Metagenomic sequences (MAG name indicated, if applicable)
Representative *mcrA* gene amplicon sequences

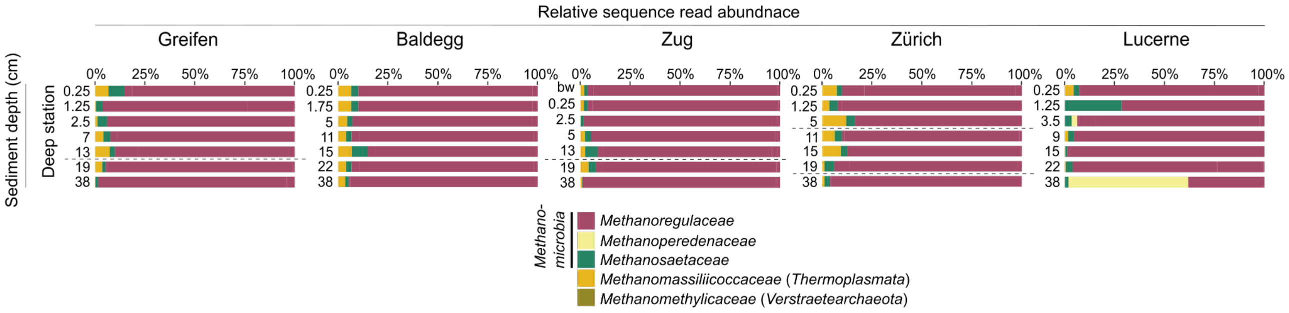

Figure S3: Relative abundances of *mcr*A gene sequences in 35 lake sediment metagenomes. *mcr*A genes of assembled metagenomic contigs were dereplicated and classified by placement in the *mcr*A phylogenetic tree. Relative abundances were determined by mapping raw sequence reads to the contigs encoding the *mcr*A genes.

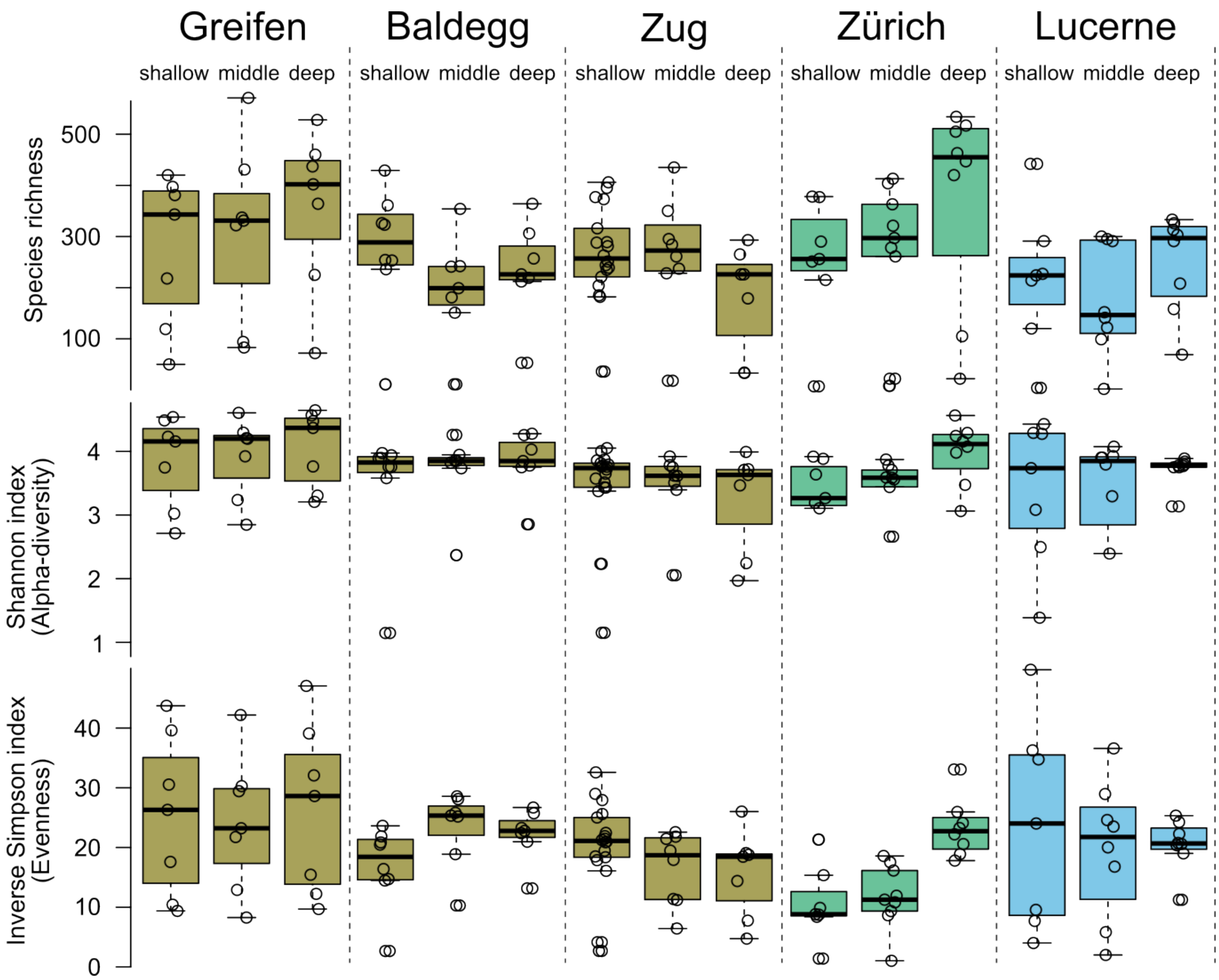

Figure S4: Diversity indices of different lakes and lake stations based on OTUs of *mcrA* gene amplicons. Note, that trophic status of the lake does not appear to affect diversity or richness of methane-cycling archaea.

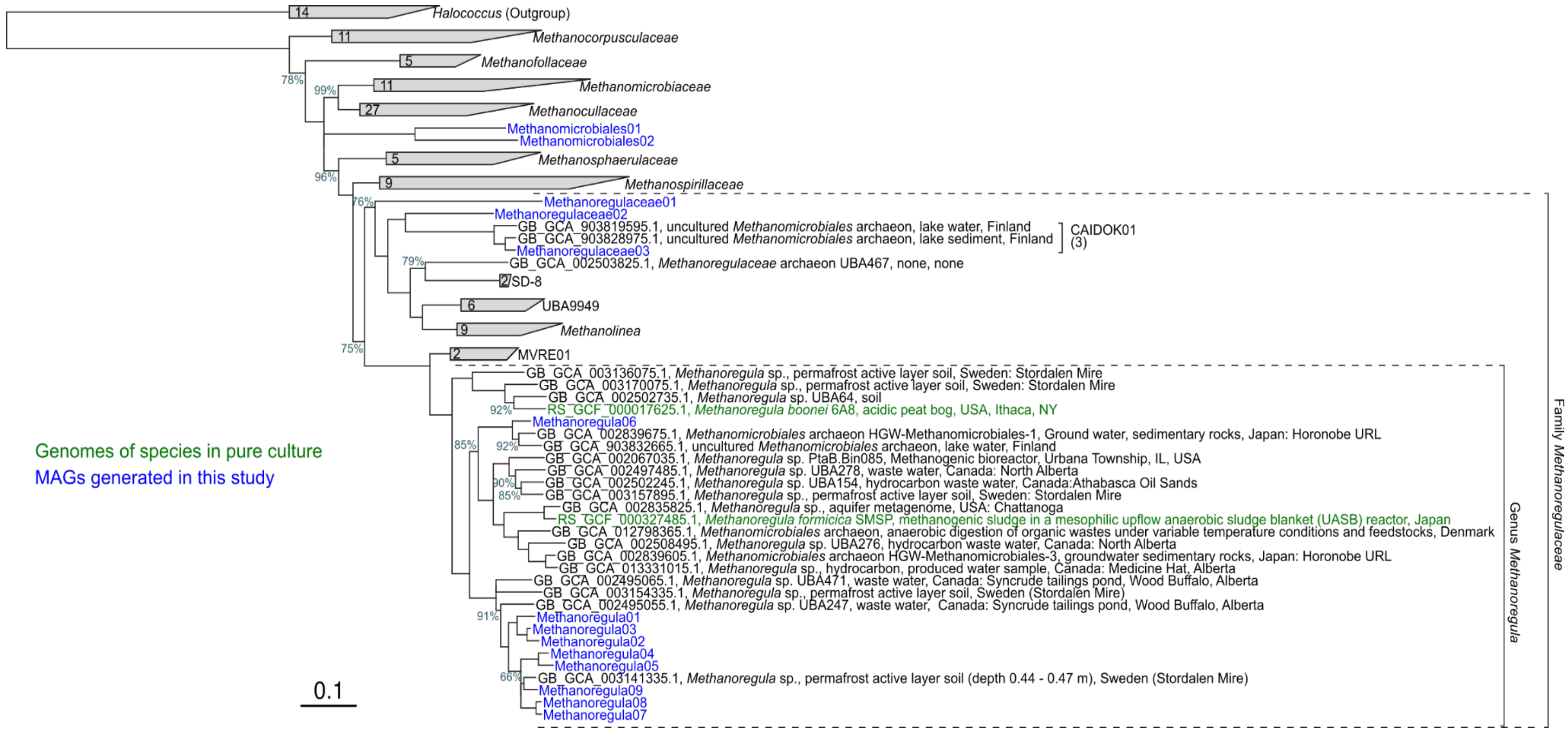

Figure S5: Phylogenomic placement of *Methanomicrobiales* MAGs in the GTDB genome tree [15]. Genomes are placed into the tree based on concatenated alignment of 120 single-copy marker proteins for classification purposes as described in [14]. Note that MAG Methanomicrobiales01 represents a first genome of a previously uncultured family corresponding to the ‘Methanomicrobiales environmental cluster I’ of *mcr*A genes (Fig. S2B).

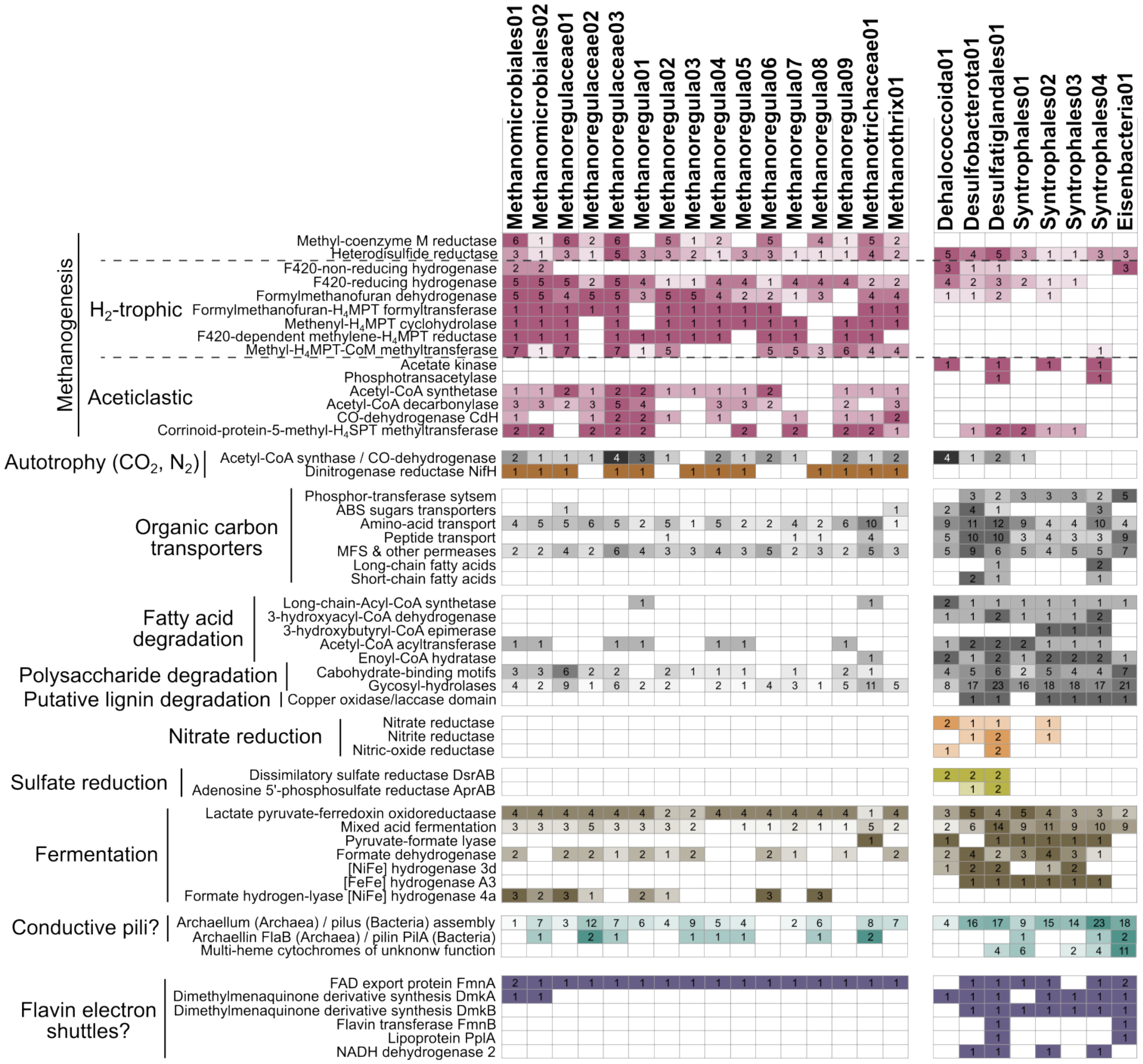

Figure S6: Genes indicative of selected metabolisms (rows) found within the MAGs of methanogens and potentially syntrophic microorganisms (columns). Numbers in the table stand for differently annotated genes within each category, e.g. encoding different subunits of the same enzyme (e.g. methyl-CoM reductase), or different enzymes involved in the same pathway (e.g. mixed-acid fermentation). For the full list of annotations see File S2.

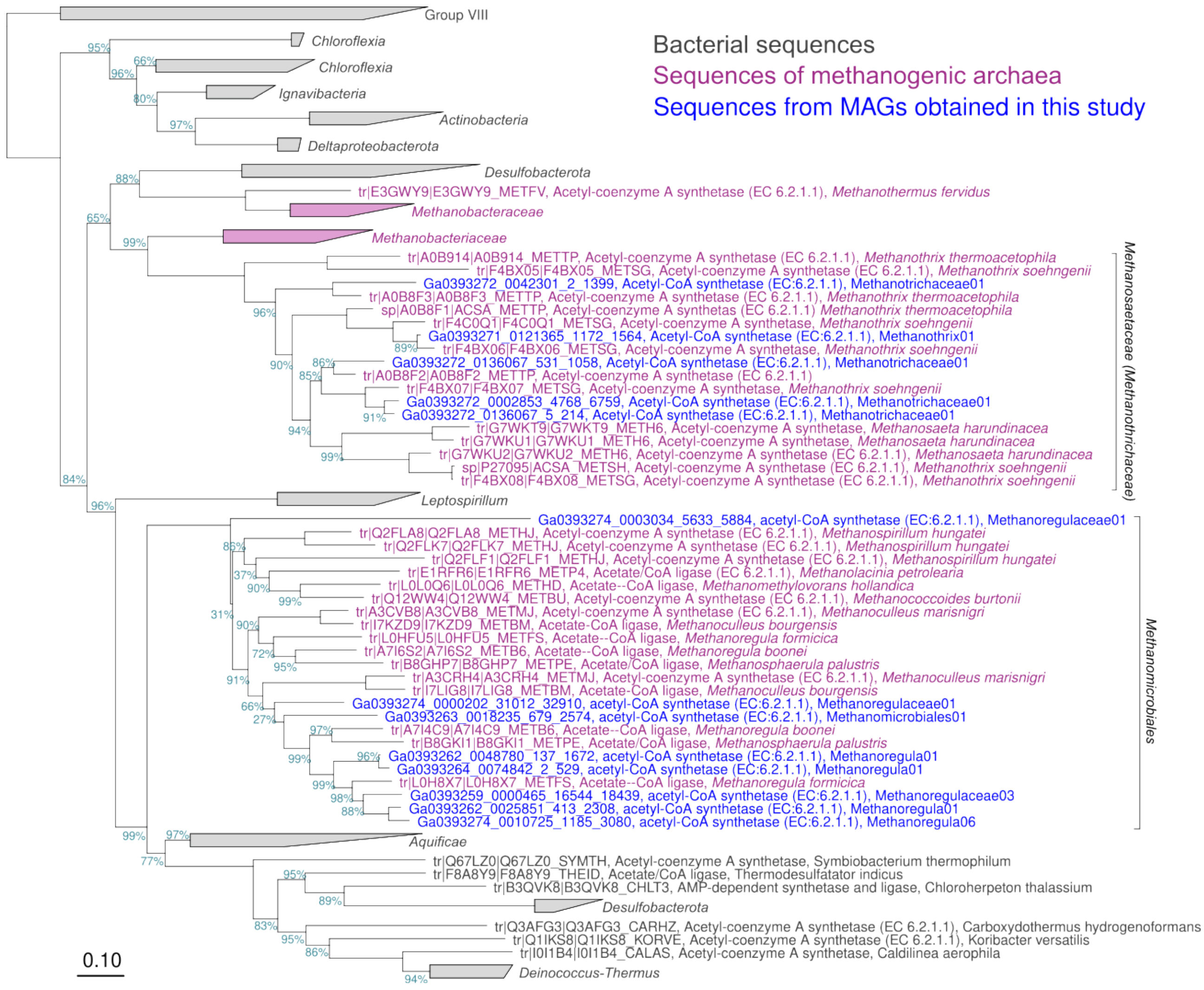

Figure S7: Phylogenetic tree of Acetyl-CoA synthetase Group VII (according to [39]). A phylogenetic tree was calculated with FastTree using the Le-Gascuel substitution model based on amino-acid positions conserved in at least 25% of the sequences according to MAFFT E-INS-I alignment. Reference sequences were obtained from UniprotKB. Note, that although genomes of hydrogenotrophic methanogens also encode an Acetyl-CoA synthetase, their version of the enzyme falls into a clade distinct from the Acetyl-CoA synthetase of aceticlastic methanogens.

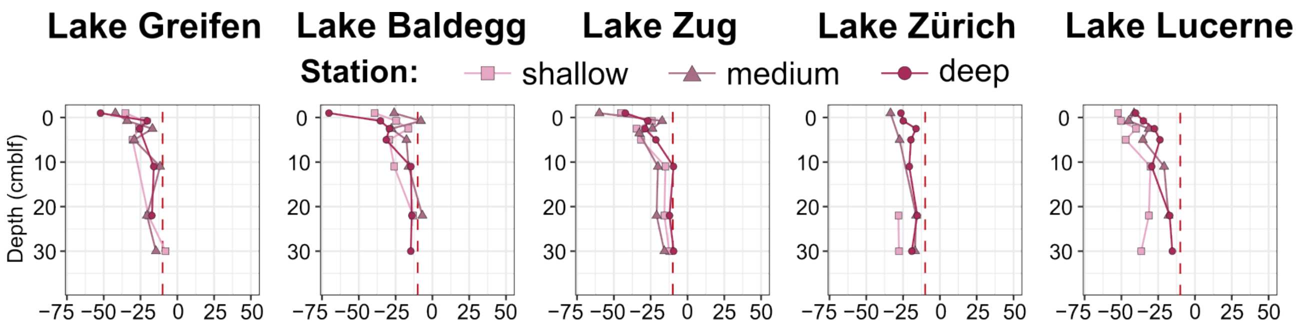

Figure S8: Gibbs energies (ΔG*r*') of anaerobic acetate oxidation (in kJ mol^{-1}). Red dashed lines indicate the biological energy quantum (-10 kJ mol^{-1}).

Supplementary References

1. Brian Bushnell. BBtools. 2017.
2. Nurk S, Meleshko D, Korobeynikov A, Pevzner PA. metaSPAdes: a new versatile metagenomic assembler. *Genome Res* 2017; **27**: 824–834.
3. Chen I-MA, Chu K, Palaniappan K, Pillay M, Ratner A, Huang J, et al. IMG/M v.5.0: an integrated data management and comparative analysis system for microbial genomes and microbiomes. *Nucleic Acids Res* 2019; **47**: D666–D677.
4. Zhang H, Yohe T, Huang L, Entwistle S, Wu P, Yang Z, et al. dbCAN2: a meta server for automated carbohydrate-active enzyme annotation. *Nucleic Acids Res* 2018; **46**: W95–W101.
5. Lombard V, Golaconda RH, Drula E, Coutinho PM, Henrissat B. The carbohydrate-active enzyme database (CAZy) in 2013. *Nucleic Acids Res* 2014; **42**.
6. Garber AI, Nealson KH, Okamoto A, McAllister SM, Chan CS, Barco RA, et al. FeGenie: A Comprehensive Tool for the Identification of Iron Genes and Iron Gene Neighborhoods in Genome and Metagenome Assemblies. *Front Microbiol* 2020; **11**: 37.
7. Kang DD, Froula J, Egan R, Wang Z. MetaBAT, an efficient tool for accurately reconstructing single genomes from complex microbial communities. *PeerJ* 2015; **3**: e1165.
8. Wu YW, Tang YH, Tringe SG, Simmons BA, Singer SW. MaxBin: An automated binning method to recover individual genomes from metagenomes using an expectation-maximization algorithm. *Microbiome* 2014; **2**: 26.
9. Alneberg J, Bjarnason BS, de Bruijn I, Schirmer M, Quick J, Ijaz UZ, et al. Binning metagenomic contigs by coverage and composition. *Nat Methods* 2014; **11**: 1144–1146.
10. Sieber CMK, Probst AJ, Sharrar A, Thomas BC, Hess M, Tringe SG, et al. Recovery of genomes from metagenomes via a dereplication, aggregation and scoring strategy. *Nature Microbiology* 2018; **3**: 836–843.
11. Parks DH, Imelfort M, Skennerton CT, Hugenholz P, Tyson GW, Hugenholtz P, et al. CheckM: assessing the quality of microbial genomes recovered from isolates, single cells, and metagenomes. *Genome Res* 2015; **25**: 1043–1055.
12. Olm MR, Brown CT, Brooks B, Banfield JF. dRep: a tool for fast and accurate genomic comparisons that enables improved genome recovery from metagenomes through de-replication. *ISME J* 2017; **11**: 2864–2868.
13. Eren AM, Esen OC, Quince C, Vineis JH, Morrison HG, Sogin ML, et al. Anvi’o: an advanced analysis and visualization platform for 'omics data. *PeerJ* 2015; **3**: e1319.
14. Chaumeil P-A, Mussig AJ, Hugenholtz P, Parks DH. GTDB-Tk: a toolkit to classify genomes with the Genome Taxonomy Database. *Bioinformatics* 2019.
15. Parks DH, Chuvochina M, Waite DW, Rinke C, Skarshewski A, Chaumeil PA, et al. A standardized bacterial taxonomy based on genome phylogeny substantially revises the tree of life. *Nat Biotechnol* 2018; **36**: 996.
16. Dombrowski N, Williams TA, Sun J, Woodcroft BJ, Lee J-H, Minh BQ, et al. Undinarchaeota illuminate DPANN phylogeny and the impact of gene transfer on archaeal evolution. *Nat Commun* 2020; **11**: 3939.
17. Price MN, Dehal PS, Arkin AP. FastTree 2 - approximately maximum-likelihood trees for large alignments. *PLoS One* 2010; **5**: e9490.

18. Le SQ, Gascuel O. An improved general amino acid replacement matrix. *Mol Biol Evol* 2008.

19. Gascuel O. BIONJ: an improved version of the NJ algorithm based on a simple model of sequence data. *Mol Biol Evol* 1997; **14**: 685–695.

20. Søndergaard D, Pedersen CNS, Greening C. HydDB: A web tool for hydrogenase classification and analysis. *Sci Rep* 2016; **6**: 34212.

21. Jones P, Binns D, Chang HY, Fraser M, Li W, McAnulla C, et al. InterProScan 5: Genome-scale protein function classification. *Bioinformatics* 2014; **30**: 1236–1240.

22. Katoh K, Standley DM. MAFFT multiple sequence alignment software version 7: improvements in performance and usability. *Mol Biol Evol* 2013; **30**: 772–780.

23. Walker DJ, Adhikari RY, Holmes DE, Ward JE, Woodard TL, Nevin KP, et al. Electrically conductive pili from pilin genes of phylogenetically diverse microorganisms. *ISME J* 2018; **12**: 48–58.

24. Walker DJF, Nevin KP, Holmes DE, Rotaru A-E, Ward JE, Woodard TL, et al. Syntrophus conductive pili demonstrate that common hydrogen-donating syntrophs can have a direct electron transfer option. *ISME J* 2020; **14**: 837–846.

25. Light SH, Su L, Rivera-Lugo R, Cornejo JA, Louie A, Iavarone AT, et al. A flavin-based extracellular electron transfer mechanism in diverse Gram-positive bacteria. *Nature* 2018; **562**: 140–144.

26. Jackson BE, Bhupathiraju VK, Tanner RS, Woese CR, McInerney MJ. Syntrophus aciditrophicus sp. nov., a new anaerobic bacterium that degrades fatty acids and benzoate in syntrophic association with hydrogen-using microorganisms. *Arch Microbiol* 1999; **171**: 107–114.

27. Sieber JR, Le HM, McInerney MJ. The importance of hydrogen and formate transfer for syntrophic fatty, aromatic and alicyclic metabolism. *Environ Microbiol* 2014; **16**: 177–188.

28. Galushko A, Kuever J. Syntrophaceae. *Bergey's Manual of Systematics of Archaea and Bacteria*. John Wiley & Sons, 2020; pp 1–3.

29. McInerney MJ, Rohlin L, Mouttaki H, Kim U, Krupp RS, Rios-Hernandez L, et al. The genome of *Syntrophus aciditrophicus*: Life at the thermodynamic limit of microbial growth. *Proceedings of the National Academy of Sciences* 2007; **104**: 7600–7605.

30. Soares R, Costa NL, Paquete CM, Andreini C, Louro RO. A New Paradigm of Multiheme Cytochrome Evolution by Grafting and Pruning Protein Modules. *Mol Biol Evol* 2022; **39**.

31. Costa NL, Hermann B, Fourmond V, Faustino MM, Teixeira M, Einsle O, et al. How Thermophilic Gram-Positive Organisms Perform Extracellular Electron Transfer: Characterization of the Cell Surface Terminal Reductase OcwA. *MBio* 2019; **10**.

32. Holmes DE, Rotaru A-E, Ueki T, Shrestha PM, Ferry JG, Lovley DR. Electron and Proton Flux for Carbon Dioxide Reduction in Methanosarcina barkeri During Direct Interspecies Electron Transfer. *Front Microbiol* 2018; **9**: 3109.

33. Gao K, Lu Y. Putative Extracellular Electron Transfer in Methanogenic Archaea. *Front Microbiol* 2021; **12**: 611739.

34. Yee MO, Rotaru A-E. Extracellular electron uptake in Methanosarcinales is independent of multiheme c-type cytochromes. *Sci Rep* 2020; **10**: 372.

35. Walker DJF, Martz E, Holmes DE, Zhou Z, Nonnenmann SS, Lovley DR. The Archaellum of Methanospirillum hungatei Is Electrically Conductive. *MBio* 2019; **10**.

36. Shock EL, Sassani DC, Willis M, Sverjensky DA. Inorganic species in geologic fluids: correlations among standard molal thermodynamic properties of aqueous ions and hydroxide complexes. *Geochim Cosmochim Acta* 1997; **61**: 907–950.

37. Wagman DD, Evans WE, Parker VB, Schumm RH, Halow I, Bailey, et al. The NBS tables of chemical thermodynamic properties: Selected values for inorganic and C1 and C2 organic substances in SI units. *J Phys Chem Ref Data* 1982; **11**.

38. Shock EL, Helgeson HC. Calculation of the thermodynamic and transport properties of aqueous species at high pressures and temperatures: Standard partial molal properties of organic species. *Geochim Cosmochim Acta* 1990; **54**: 915–945.

39. Amend JP, Shock EL. Energetics of overall metabolic reactions of thermophilic and hyperthermophilic Archaea and bacteria. *FEMS Microbiol Rev* 2001; **25**: 175–243.

40. Ingram-Smith C, Smith KS. AMP-forming acetyl-CoA synthetases in Archaea show unexpected diversity in substrate utilization. *Archaea* 2007; **2**: 95–107.

41. De Brabandere L, Thamdrup B, Revsbech NP, Foadi R. A critical assessment of the occurrence and extent of oxygen contamination during anaerobic incubations utilizing commercially available vials. *J Microbiol Meth* 2012; **88**:147-154.
